# The molecular architecture of the nuclear basket

**DOI:** 10.1016/j.cell.2024.07.020

**Published:** 2024-08-09

**Authors:** Digvijay Singh, Neelesh Soni, Joshua Hutchings, Ignacia Echeverria, Farhaz Shaikh, Madeleine Duquette, Sergey Suslov, Zhixun Li, Trevor van Eeuwen, Kelly Molloy, Yi Shi, Junjie Wang, Qiang Guo, Brian T. Chait, Javier Fernandez-Martinez, Michael P. Rout, Andrej Sali, Elizabeth Villa

**Affiliations:** 1 School of Biological Sciences, University of California, San Diego, La Jolla, CA 92093, USA; 2 Department of Bioengineering and Therapeutic Sciences, University of California, San Francisco, San Francisco, CA 94158, USA; 3 Department of Cellular and Molecular Pharmacology, University of California, San Francisco, San Francisco, CA 94158, USA; 4 State Key Laboratory of Protein and Plant Gene Research, Peking-Tsinghua Center for Life Sciences, Academy for Advanced Interdisciplinary Studies, School of Life Sciences, Peking University, Beijing 100871, P.R. China; 5 Laboratory of Cellular and Structural Biology, The Rockefeller University, New York, NY 10065, USA; 6 Laboratory of Mass Spectrometry and Gaseous Ion Chemistry, The Rockefeller University, New York, NY 10065, USA; 7 Ikerbasque, Basque Foundation for Science, 48013 Bilbao, Spain; 8 Instituto Biofisika (UPV/EHU, CSIC), University of the Basque Country, 48940 Leioa, Spain; 9 Quantitative Biosciences Institute, University of California, San Francisco, San Francisco, CA 94158, USA; 10 Department of Pharmaceutical Chemistry, University of California, San Francisco, San Francisco, CA 94158, USA; 11 Howard Hughes Medical Institute, University of California, San Diego, La Jolla, CA 92093, USA; 12 These authors contributed equally; 13 Present address: Thermofisher Scientific, Waltham, MA, USA; 14 Present address: Department of Pharmacological Sciences, Icahn School of Medicine at Mount Sinai, 1425 Madison Avenue, 16-78B, New York, NY 10029, USA; 15 Lead contact

## Abstract

The nuclear pore complex (NPC) is the sole mediator of nucleocytoplasmic transport. Despite great advances in understanding its conserved core architecture, the peripheral regions can exhibit considerable variation within and between species. One such structure is the cage-like nuclear basket. Despite its crucial roles in mRNA surveillance and chromatin organization, an architectural understanding has remained elusive. Using in-cell cryo-electron tomography and subtomogram analysis, we explored the NPC’s structural variations and the nuclear basket across fungi (yeast; *S. cerevisiae*), mammals (mouse; *M. musculus*), and protozoa (*T. gondii*). Using integrative structural modeling, we computed a model of the basket in yeast and mammals that revealed how a hub of nucleoporins (Nups) in the nuclear ring binds to basket-forming Mlp/Tpr proteins: the coiled-coil domains of Mlp/Tpr form the struts of the basket, while their unstructured termini constitute the basket distal densities, which potentially serve as a docking site for mRNA preprocessing before nucleocytoplasmic transport.

## In brief

The elusive architecture of the nuclear basket reveals that a double ring is necessary to stabilize a hub of Nups from which the struts emanate. The basket’s distal density contains the N/C termini of the strut-forming proteins that serve as a docking platform for cargo and nuclear periphery elements.

## Graphical abstract

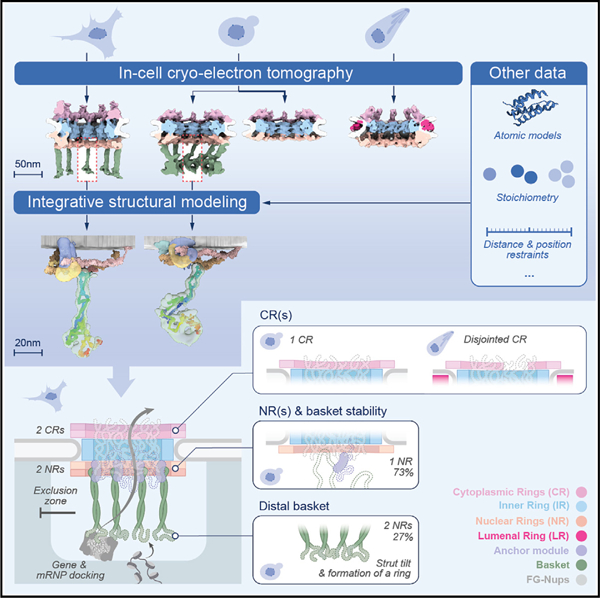

## INTRODUCTION

The nuclear pore complex (NPC) is a massive macromolecular assembly in the nuclear envelope (NE), responsible for nucleocytoplasmic transport.^1–4^ It comprises hundreds of proteins of more than 30 different types, known as nucleoporins (Nups). These Nups, present in copies ranging from 8 to 48, assemble into multiple rings stacked along the NE.^1–10^ These include outer rings on the nuclear and cytoplasmic sides (nuclear ring [NR] and cytoplasmic ring [CR]) and an inner ring (IR) located between them. Each ring generally consists of 8 repeating subunits. Phenylalanine-glycine (FG)-rich repeats present in multiple Nups emanate inward from these rings to form the NPC’s central channel and interact with transport factors to enable nucleocytoplasmic transport.^5,11^ Apart from the CR, IR, and NR, the NPC features another prominent module known as the nuclear basket, also referred to as the basket.^12–18^ The basket is believed to play roles in mRNA transport and chromatin organization.^14,18–24^ In yeast, Nup1, Nup2, Nup60, and Mlp1/Mlp2 are the main components of the basket and are referred to as basket Nups.^4,24–26^ Their mammalian counterparts include Nup153 (ortholog of Nup60), Nup50 (ortholog of Nup2), and Tpr (ortholog of Mlp1/2).^12,13^ The basket has been observed in atomic force microscopy, electron microscopy (EM), and in cryo-electron tomography (cryo-ET) studies of biochemically isolated NEs and NPCs of many organisms,^15–17,24,27,28^ although the exact role of different Nups in the observed basket structures is not well defined. In these studies, the basket is described as an assembly consisting of eight struts emanating from the nuclear side of the NPC core that converge into distal densities that could restructure and dilate to allow passage of large cargoes through the NPC.^20^ These studies also highlighted the need for further studies, including obtaining a 3D map of the basket in-cell and describing the molecular organization of basket Nups, along with exploring the structural dynamics of the basket and its neighboring peripheral NPC structures in-cell.

While the major observable features of the NPC, chiefly the basket and rings, have been known for decades in vertebrates, their organization and variability—from within a single cell to between species—have been largely undefined. However, recent work has highlighted that the NPC’s architecture may vary significantly both within and between species.^5,29^ To explore the nature of such variations, we performed in-cell cryo-ET on NPCs of cells from three different and evolutionarily divergent eukaryotes: fungi (*S. cerevisiae*), mammals (*M. musculus*; mouse’ National Institute of Health 3-day transfer, inoculum 3 × 10^5 [NIH3T3] cells), and parasitic protozoa (*T. gondii*) (Figure S1A). The cells of these organisms were rendered amenable to in-cell cryo-ET through cryo-focused ion beam (FIB) milling,^30–32^ that produces lamellae thin enough for cryo-ET from vitrified cells.^33^ Our in-cell cryo-ET dataset represents one of the largest of its kind, comprising 1,604 tilt series. Through subtomogram analysis and 3D classification, we classified different variants of NPCs across these organisms, yielding maps of one mammalian NPC (mNPC), one parasitic NPC (pNPC), and two distinct yeast NPC (yNPC) variants in-cell. The prefixes “m,” “p,” and “y” have been used to denote mammals (mouse; *M. musculus*), parasitic protozoa (*T. gondii*), and yeast (*S. cerevisiae*), respectively. From these maps, we discerned the basket architecture in the mNPC and one yNPC variant, shedding light on how the basket is organized on the NPC. Subsequently, we performed integrative structural modeling, incorporating a vast array of biochemical data on basket Nups along with our in-cell maps, to model the molecular architecture of the basket Nups. These maps and models provided us with a structural blueprint of how the basket forms and functions within the NPC.

## RESULTS

### A stable basket is associated with a double NR

**Figure 1.**
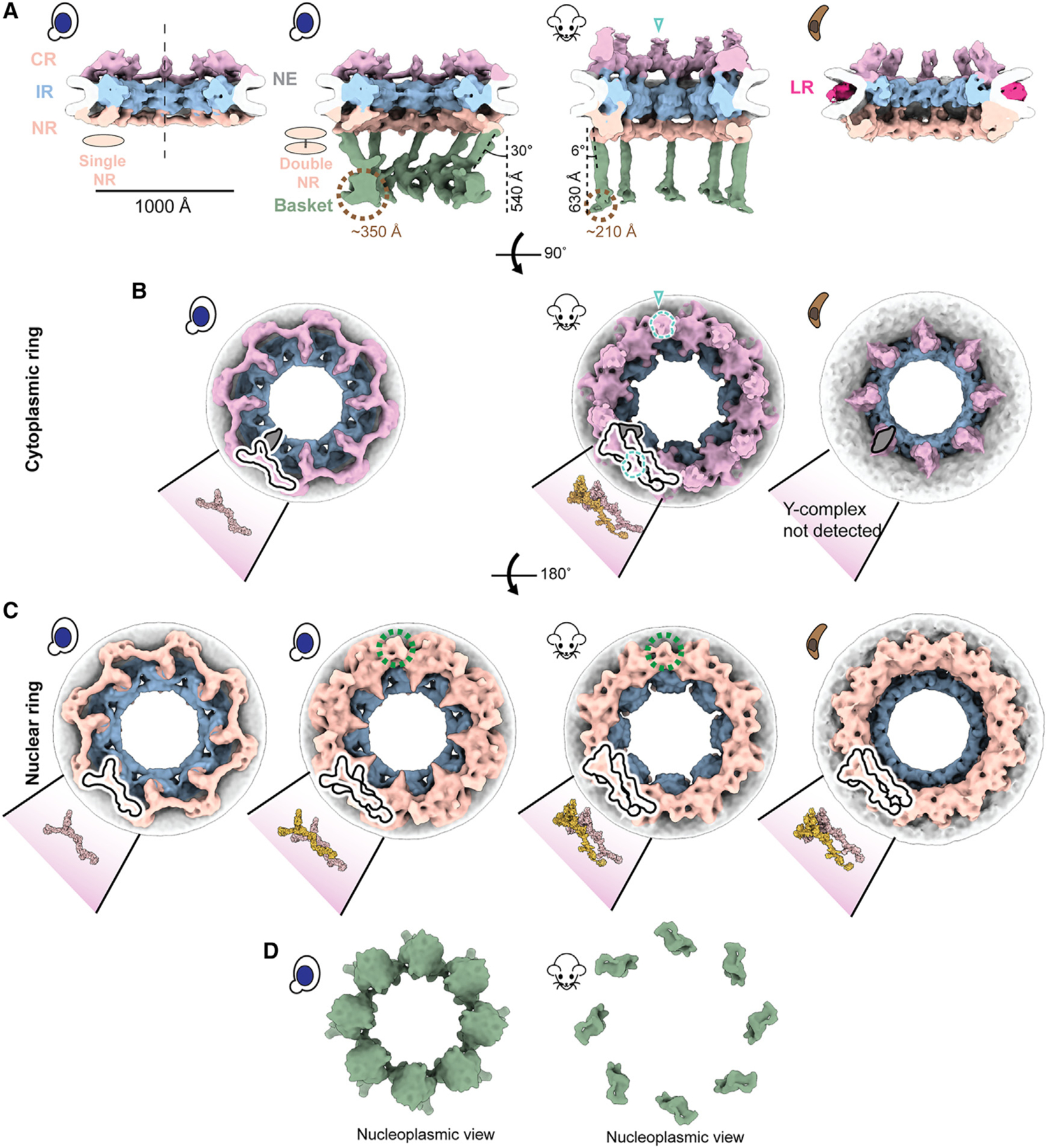
A stable nuclear basket is bound to a double nuclear ring (A) Cross-sectional views along the central axis (dashed line) of the in-cell cryo-ET maps of yNPC with single and double NR variants, mNPC and pNPC. The nuclear basket is resolved for the mNPC and yNPC with a double NR. The location of cytoplasmic filaments for the mNPC CR are indicated by teal open triangles in (A) and (B). (B) Cytoplasmic views of the CR of yNPC with a single CR, mNPC with a double CR, and pNPC with an incomplete CR. Also depicted are models of the single or double Y-complex and the adjoining mRNA export platform (highlighted in the gray schematic), whose eight copies are arranged in head-to-tail orientation around the central axis to form the CR. Shown in dashed teal lines is the region from which the cytoplasmic filaments emanate. (C) Nucleoplasmic view of the single and double NR of yNPC and double NR of mNPC and pNPC along with the models of the single or double Y-complex. Shown in dashed green lines is the region in double NR from which the basket’s struts emanate. The models for yeast and mammalian CR/NR are from the PDB IDs: yNPC, PDB: 7N9F; mNPC, PDB: 7R5J. (D) Nucleoplasmic views of the yeast and mammalian basket. CR, cytoplasmic ring; IR, inner ring; NR, nuclear ring; NE, nuclear envelope; LR, lumenal ring. See also Figures S1 and S2.

Our previous study revealed at least two populations of yNPCs within each cell: a major population with a single nuclear NR, and a lesser population carrying a double NR.^5^ We set out to further investigate the structural variants of NPCs in yeast in-cell using cryo-ET (Figure S1A; STAR Methods). Through 3D classification of a large dataset of in-cell NPCs, we found that ~73% of NPCs in yeast during its log-phase growth possess a single NR, while the remaining have a double NR, which is consistent with the proportions estimated from quantitative fluorescence imaging (Figures 1A–1C, S1B, and S1C).^5^ The CR consists of eight subunits, each comprising one or two Y-complexes arranged in a head-to-tail orientation around the central axis passing through the center of the NPC^5,6,8,34–36^ (Figures 1B and 1C). In addition to Y-complexes, the CR also has an mRNA export platform^5,6,8,36–38^ (Figure 1B).

The single NR also consists of eight Y-complexes, while the double NR carries sixteen such complexes in its two rings; the Y-complex rings proximal to and distal from the IR are referred to as the proximal and distal NR, respectively. The classification of the yNPCs into the single and double NR variants and their subsequent refinements gave us a more homogenous map of the single NR (devoid of any double NR densities). To date, a single NR is only observed in yNPCs (Figures 1A and 1C). These single and double NR variants differ only in their NR, while their IR and CR are similar in stoichiometry and physical dimensions (Figure S1B). Notably, a basket was observed only in the double NR variant in a dataset of more than 5,100 NPC particles across 1,449 tomograms from yeast, prompting the question of whether the single NR could support a stable and stoichiometric basket (Figure 1A). However, it has previously been shown that most yNPCs, including both single and double NR forms, co-localize with basket components, with the exception of a class found only adjacent to the nucleolus that lacks Mlp1/2,^23,24^ and that basket components in yeast are generally dynamic.^5,24,39–41^ Thus, we infer that basket components are more dynamic and so less well resolved in single NR NPCs.^5,39^ Given that the basket was only resolved in the double NR variant in yeast, we wondered if the mNPC, which always has a double NR^36^ also has a discernible basket emanating from the double NR in a pattern similar to yNPC. Indeed, we also determined the architecture of the basket in mNPC and found it emanating similarly from its double NR (Figures 1A and 1C).

### A pNPC has a double NR and an incomplete CR

After observing the varying stoichiometries of the outer rings, we explored the diversity of these stoichiometries by performing in-cell cryo-ET and subtomogram analysis on NPCs of the protozoan parasite *T. gondii*, which diverged at least 1.5 billion years ago^42^ and is responsible for toxoplasmosis in humans.^43^ Surprisingly, this protozoan NPC (pNPC) features a double NR but has a minimal CR which is discontinuous, even more so than the disjointed CR observed in the fungi *S. pombe* (Figures 1A–1C and S2).^44^ Focused refinement of the pNPC’s subunits revealed that the incomplete CR appears to lack a full-length Y-complex, which cannot be fully rationalized due to the lack of biochemical data about pNPC (Figure 1B). Notably, pNPC has the smallest diameter observed so far under normal, i.e., non-stress and in-cell conditions. Its lumenal ring (LR) is more prominent, unlike the LR of yNPC and mNPC, which are closer to the NE and thus more difficult to resolve (Figure 1A). The LR of the NPC separates from the NE upon the NPC’s contraction and becomes distinctly more visible, as was also observed for contracted isolated yNPCs and yNPCs in-cell under cellular stress.^5,44^

### The basket consists of struts emanating from a double NR that end in a distal globular density

The basket consists of eight struts, each ~100 Å thick, emanating from each of the eight subunits of the double NR at an angle from the central axis (30° for yBasket and 6° for mBasket), and terminating in a globular density referred to as the basket distal density (or basket ring), which is 540 and 630 Å away from the double NR in the yBasket and mBasket, respectively (Figure 1A). The significant inward projection of the struts in the yBasket toward the central axis of the NPC is consistent with projections observed for the yBasket in EM studies of isolated yeast NEs.^17,24^ The size of a single (one out of eight) basket distal density is ~350 Å for the yBasket and ~210 Å for the mBasket (Figure 1A). In the yBasket, these densities are connected to form a ring with a diameter of ~760 Å. However, no connections between distal densities were resolved in the mBasket with an apparent distal diameter of ~1,000 Å (Figures 1A and 1D). These features of the basket bear broad similarities to those identified in isolated NEs and nuclei from various species,^15,16,24,27^ except that the basket distal densities in isolated samples were mostly found to be connected, with their ring contracted, unlike those in our in-cell maps (Figures 1A and 1D).^15,16,24,27^

### The nuclear periphery exhibits an exclusion zone around the basket

**Figure 2.**
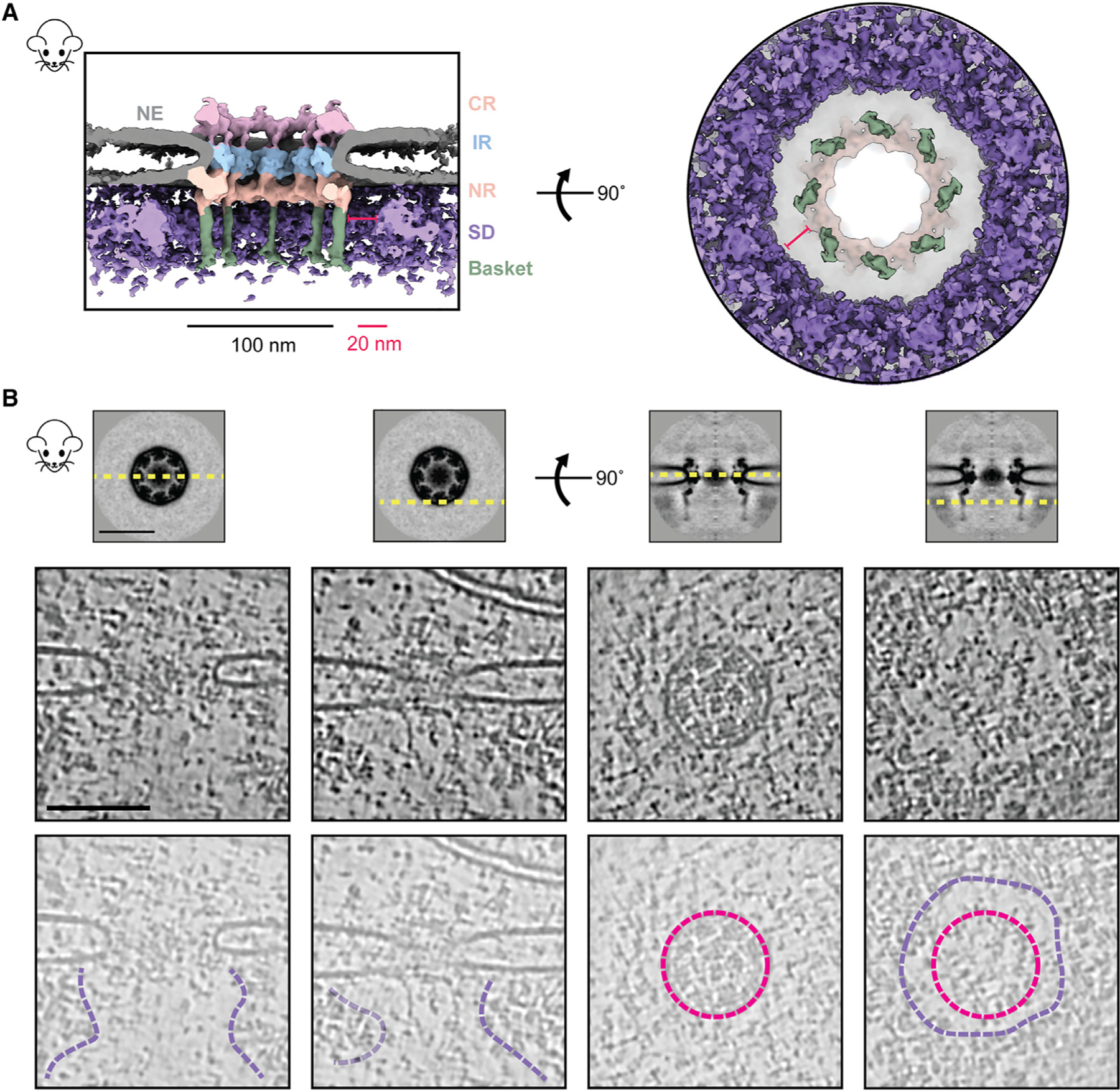
Direct observation of the heterochromatin exclusion zone around the mNPC (A) The cross-sectional (left) and nucleoplasmic view (right) of the C8-symmetrized average map of mNPC shows that molecular crowding (via surrounding densities [SDs]) around the mNPC is absent in the immediate vicinity of the basket (~20 nm). (B) Tomogram slices of different regions around the NPC show the extent of this exclusion zone. Top: slices of the mNPC average used to depict viewing planes (yellow, dashed) in tomogram slices below. Middle and bottom: tomogram slices of an mNPC viewed parallel (left) and perpendicular (right) to the plane of the NE, as depicted in the top panel. Tomogram slices are duplicated in the bottom row to show annotated views. Lumenal rings (pink) and boundaries of the exclusion zone (purple) are indicated. CR, cytoplasmic ring; IR, inner ring; NR, nuclear ring; NE, nuclear envelope; SDs, surrounding densities. Scale bars, 100 nm unless stated otherwise. See also Figure S3.

We leveraged the advantage of in-cell cryo-ET to examine the molecular environment in the proximity of NPCs with single and double NRs to explore why *S. cerevisiae* has two structural variants in NPCs and whether these variants had some specialized spatial distribution in the nucleus (Figures S3A and S3B).^33,45^ In contrast to our expectation, in many cases, yNPCs with either a single or a double NR (those with a stable basket) were nevertheless found in similar environments, often adjacent to each other (Figures S3A and S3B). One proposed role of the basket is to help create an exclusion zone around the NPC, presumably to streamline nucleocytoplasmic transport.^21,24^ This hypothesis arose from observations of fixed and stained specimens in 2D electron micrographs.^21,24^ Here, we generated a 3D average of the nucleoplasmic densities around the NPC to more clearly observe this zone and contextualize it with the basket structure. We used mNPCs, as they have morphologically better-defined heterochromatin than yeast (Figure S3C). In individual tomograms, we could directly see a well-defined exclusion zone of ~20 nm surrounding the nuclear basket (Figure 2B). To establish if this was a general feature, we generated a map of the mNPC with a much larger box size, encompassing a significant portion of its surroundings, including nucleoplasmic regions (Figure 2A). The map indeed revealed an exclusion zone around the mNPC and its basket on the nucleoplasmic side. Beyond the exclusion zone on the nucleoplasmic side, the densities likely represent lamina and chromatin, supporting the role of the NPC in chromatin organization.^46,47^

### The molecular architecture of the yeast and mammalian baskets

**Figure 3.**
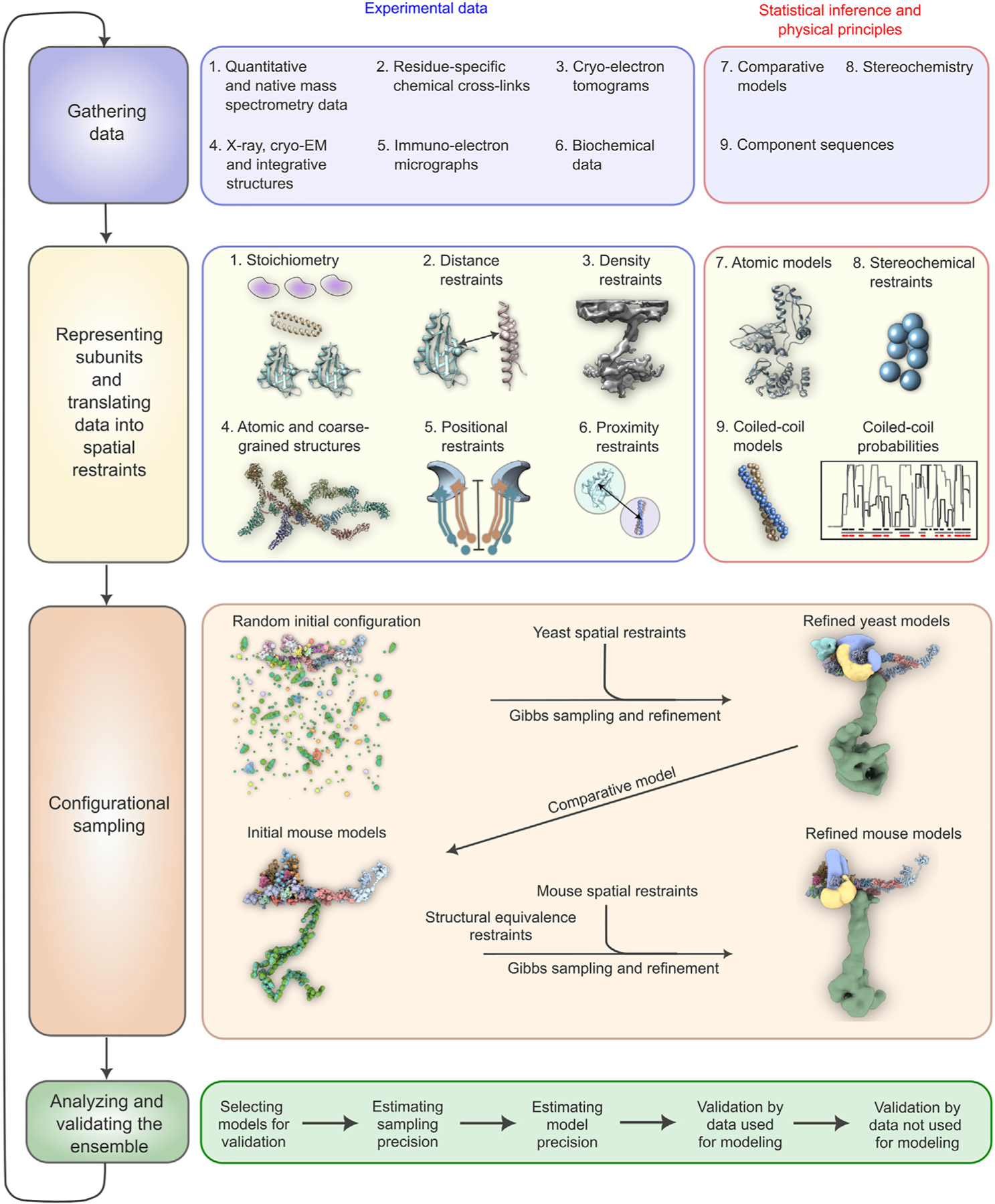
The four-stage scheme for integrative modeling of the baskets Our integrative approach proceeds through four stages: (1) gathering data, (2) representing subunits and translating the data into spatial restraints, (3) configurational sampling to produce an ensemble of models that satisfies the restraints, and (4) analyzing and validating the ensemble. Stage 1 lists the experimental information used in this study for integrative modeling of baskets of yeast and mammals. Stage 2 lists representation and extracted spatial restraints obtained from information gathered in stage 1. Stage 3 describes the configurational sampling to search for the models that satisfy the input information. A Gibbs sampling starting from a random initial configuration for the yBasket Nups generates the ensemble of good scoring models. The centroid model of the yBasket ensemble was used to model an initial mammalian basket. A similar Gibbs sampling with additional restraints from the mammalian data and structural equivalence restraint generates the ensemble of good scoring models. Stage 4 lists the model selection and validation protocol for the ensemble of good scoring configuration for both yeast and mammals. See also Figure S4.

We used our iterative four-stage integrative approach to produce architectural maps of the yeast and mammalian baskets (Figure 3; STAR Methods),^6,48–52^ based on subunit structure models, cryo-ET maps, chemical crosslinks, immuno-EM, coiled-coil propensities, sequence connectivity, excluded volume, and published data (Figure 3, stages 1 and 2; Table S1), and a similar approach was used previously to determine the structure of the entire NPC.^6^ The modeling of the yBasket included basket Nups (yMlp, yNup1, yNup60, and yNup2, without their FG repeats) and NR Nups (yNup84 complexes), while the mammalian model consists of the basket Nups mTpr, mNup50, mNup153, and the NR mNup107 complexes (Figure S4; Tables S2 and S3). The model optimizes the conformations and positions of these components while keeping the yNup84/mNup107 complexes fixed in their previously identified locations within our maps (Figure 3, stage 3; Tables S2 and S3). Before interpreting the models, we validated them using our standard assessment process (Figure 3, stage 4; Tables S2 and S3; STAR Methods).^50,52^ Both models satisfy the data used to construct them (Figure 3, stages 1, S5, and S6). In particular, the key input information, including the cryo-ET map, chemical crosslinks, coiled-coil propensities, and subunit structure models, is satisfied by a single cluster of structural solutions with an overall precision of 57 and 42 Å for yeast and mammalian, respectively (Figures 3, S5, and S6; Tables S2 and S3); the model precision is defined as the variability of the good scoring solutions quantified by the average root-mean-square deviation (RMSD) of all solutions in the cluster. These precision estimates are considered when analyzing model features and comparing the two basket models.

### The yeast and mammalian basket models are similar in topology but different in their overall shape

**Figure 4.**
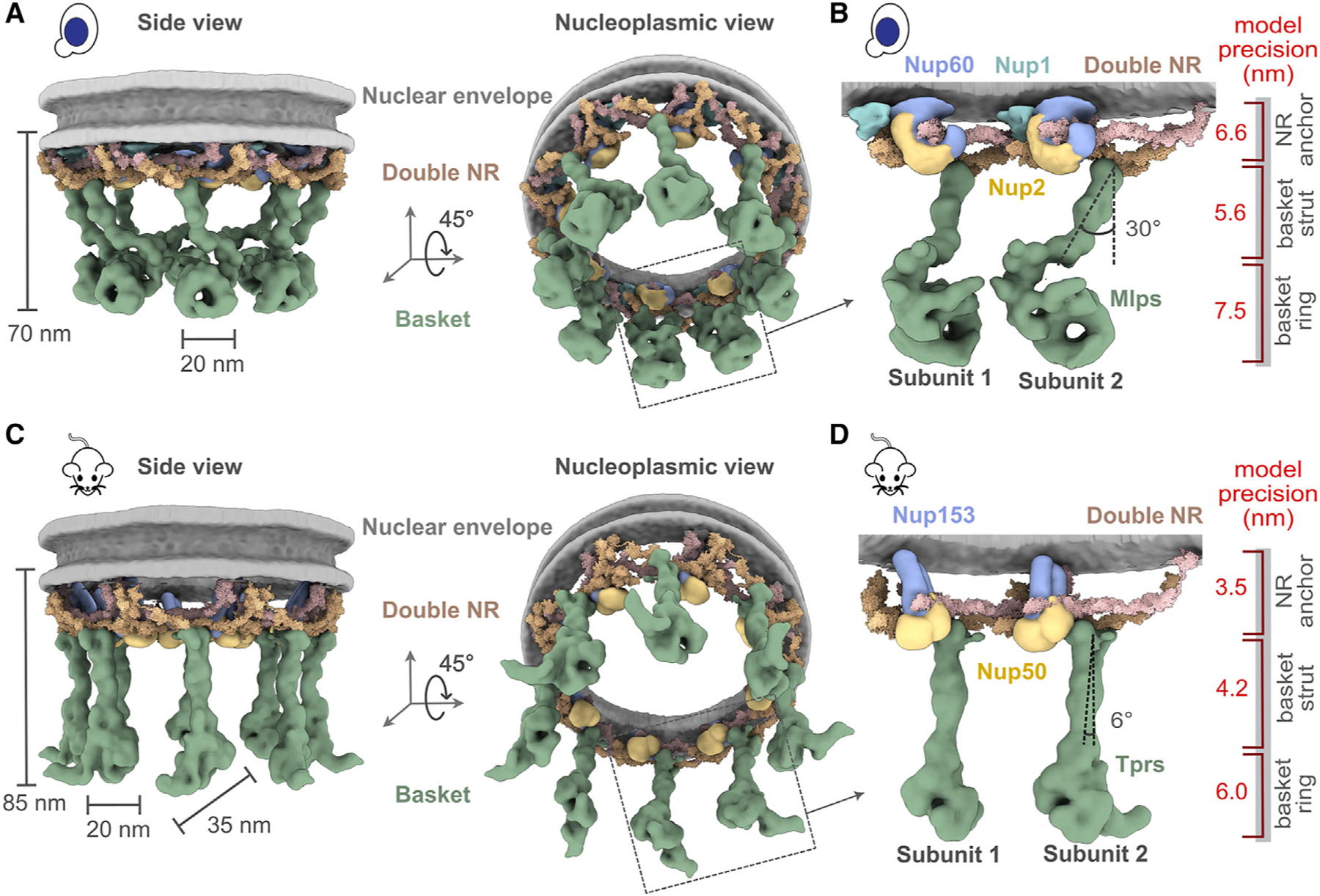
Integrative structure models and precisions of yeast and mammalian nuclear baskets (A and C) Localization probability density for the yBasket (containing yMlp, yNup1, yNup60, and yNup2 Nups) and mBasket (containing mTpr, mNup50, and mNup153 Nups) obtained from the ensemble of good scoring models. A localization probability density map for a set of models is defined as the probability of observing a model component at any point in space. The yMlps/mTprs (green) are attached to the double NR and NE (light gray half-toroid) with a common interacting FG Nups yNup60/mNup153 (violet). (B and D) A close-up view of the two subunits of the yBasket and mBasket with model precision for different basket segments. Shown here are yMlp/mTpr (green), FG Nups yNup1 (cyan), yNup2/mNup50 (yellow), and yNup60/mNup153 (violet). See also Figures S5 and S6.

**Figure 5.**
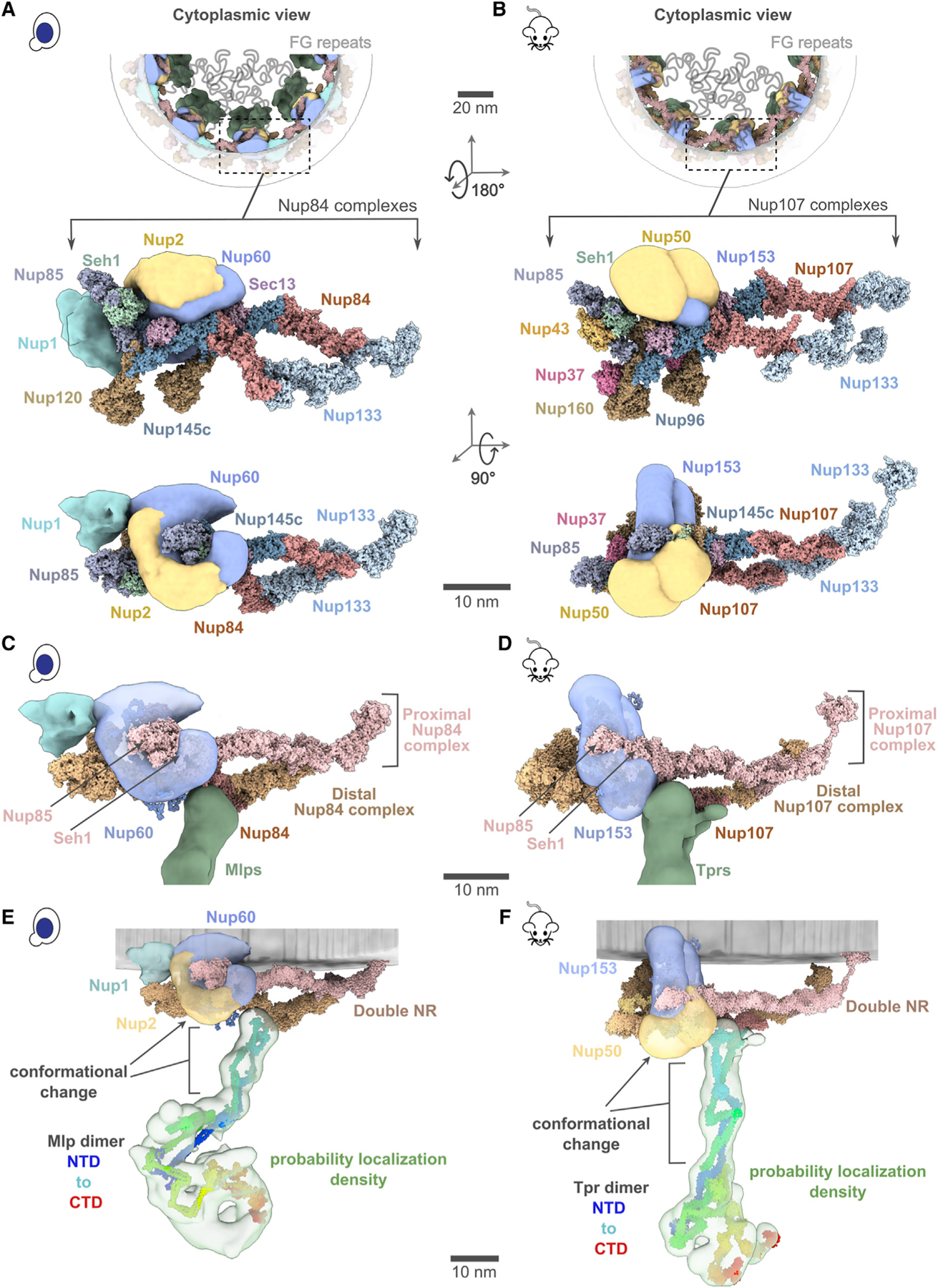
Position of different nucleoporins in the basket model (A and B) Cytoplasmic view of the yeast and mammalian basket zooms into a Y-complexes (yNup84 complex for yeast and mNup107 complex for mammalian). Schematic representations of the FG repeats and anchoring positions are shown as curly lines (gray). Each double Y-complex’s zoomed image highlights individual Nups with two different views. Localization densities of the basket anchors, yNup1 (cyan), yNup2/mNup50 (dark yellow), and yNup60/mNup153 (violet), are shown relative to the Y-complex Nups. (C and D) Close-up inner view of the localization densities of the yNup60/mNup153 (violet) that contacts with the proximal nuclear ring (pink), whereas the localization densities of yMlp/mTpr dimer (green) contacts the distal nuclear ring (tan) of the NPC. yNup2/mNup50 not shown in this view. (E and F) The yMlp/mTpr dimer centroid models were colored from the N terminus (blue) to the C terminus (red) and shown embedded within their localization probability density (light green). The region identified by the unidirectional arrows and the square brackets indicates the local conformational change of yMlp/mTpr and yNup60/mNup153 between yBasket and mBasket models. Scale bars, 20 and 10 nm. See also Figures S5 and S6.

The yeast and mammalian basket models have almost identical orthologous protein compositions (Tables S2 and S3). The mammalian model was calculated to resemble the yeast model as much as possible while satisfying all the available mammalian data (Tables S2 and S3; STAR Methods). The two models share a similar topological arrangement, albeit with notable differences in the overall shape of the basket (Figures 4A and 4C). To facilitate comparison, we dissected the basket into three modules, including the NR anchor (yNup1, yNup2, and yNup60 for yeast; mNup50 and mNup153 for mammals), basket strut (yMlp; mTpr), and basket distal modules (yMlp; mTpr) (Figures 4B and 4D). The model revealed the proximity of the NR anchor module to the central channel of the NPC, NE, and NR (Figure 4) and their association with the proximal NR via yNup60/mNup153 (Figures 5A–5D).^24^ The N terminus (blue) and C terminus of yMlp/mTpr (red) are situated in the basket distal density, while the intervening region extends toward the double NR, forming the basket strut module.

Any points regarding our modeling results and its connection with the existing literature did not use the given literature or its data as input for the modeling unless explicitly noted. The yMlps/mTprs interact with the distal NR via yNup84/mNup107, consistent with the demonstrated requirement of yNup84 for anchoring Mlps onto NPCs (Figures 5E and 5F).^24^ The presence of two binding sites for the basket Nups, one on each proximal and distal NR, places the yNup60/mNup153 and yMlps/mTprs in direct interaction. This stabilizing interaction is feasible only in the context of the double NR, highlighting its importance in assembling a stable and less dynamic basket structure. However, our model also highlights how the basket might assemble in a single NR, as yMlps and yNup60 can interact with NR proteins (yNup84, yNup85, and ySeh1) independently (Figures 5A–5D). Given the estimated model precision, our models are consistent with previously reported interactions between Nup60 and Nup2 (via their Nup60^N2BM^ domain) and Nup60 and Mlps (via their Nup60^MBM^ domain).^26,53,54^ The interaction between Nup60 and Nup2 was used as a restraint in building the models, while the interaction between Nup60 and Mlps was not. The model also revealed the presence of the coiled-coil domains of yMlp/mTpr in the basket strut module (Figures 5E and 5F). The rod-like basket strut module exhibited around a 20° tilt between the yeast and mammalian baskets, resulting in a relatively large difference in the radius of the ring formed by the basket distal density (Figure 4).^26^ The basket distal module in both baskets is approximately globular, occupying the distal end of the basket (Figures 4B and 4D). As is generally the case, it is not possible to unequivocally determine whether the differences between the yeast and mouse basket models reflect the differences between species, experimental conditions (e.g., cross links from isolated NPCs), and/or functional states.

### The FG regions in the NR anchor module face the central channel

The yNup2 had not been included in the previous model of the NPC due to the lack of data about their positions.^5,6,48^ In our models, the non-FG regions of FG Nups in the NR anchor module localize between the two copies of Nup85 in the proximal and distal yNup84/mNup107 complexes, with a precision of 6.6 nm (Figures 4B, 4D, 5A, and 5B). Both copies of yNup2 and yNup60 (and their mammalian orthologs) are proximal to each other as well as to several Nups in the double NR (Figures 5A and 5B). The yNup1 is also positioned near yNup60,^6^ anchoring both to the NE, consistent with previous mapping.^53^ This arrangement exposes the NR anchor module to the central channel. By virtue of the position of the globular anchor domain of the FG Nups, the FG anchoring sites are positioned such that the FG repeats face into the central channel, as is the case for previously localized FG repeat regions.^5,6,55^

This observation serves as further validation of our models, as this feature was not imposed on the models (Figures 5A and 5B). The anchors are not sufficiently long to extend far into the struts or into the distal basket, indicating that the associated FG repeats remain localized to the proximal end of the basket, thus spatially segregating transport from the initial docking processes occurring at the distal basket.^56,57^

## DISCUSSION

The array of stoichiometries we observe for the outer rings (CR + NR) across and within species, ranging from incomplete to 1 to 2, highlights the NPC’s modular construction and its structural plasticity, which allows it to easily adapt to gain or lose additional subcomplexes, presumably to confer alternate functionalities. *T. gondii*’s NPC appears to have the full mRNA export platform but an incomplete CR, suggesting that the full Y-complex in the CR might be more dispensable than the mRNA export platform. While *T. gondii*’s CR is quite distinct, its IR is similar to other IRs, consistent with the differences among NPCs of various organisms being more pronounced in their outer rings rather than the IR, which appears to represent the most structurally and evolutionarily conserved module of the NPC.^58^ The NPCs with alternative stoichiometries of rings can be used to understand how the NPC’s parts are formed (e.g., we show here how the distal NR could help bind and stabilize the basket) and their dispensability (e.g., an NPC can function without a full-length Y-complex on its CR, as shown here for *T. gondii* and has been shown for *S. pombe*).^44^ The most minimalist and incomplete CR observed so far in the pNPC likely contains only the mRNA export platform (possibly without the Y-complex as discussed above) or the remnant core of the Y-complex. This minimalism can be used to characterize the structural segments in the CR and Nups that are more critical for the CR’s function compared with those that are absent. Like the CR, the NR can also show different copy numbers. However, in the organisms examined so far, there is at least one complete NR as a necessary minimum for NPCs.

Beyond this, many organisms can carry two NRs on either some or all of their NPCs; and *Dictyostelium* NPCs may usually carry 3 such NRs.^59^ The reason for this variability in NR copy number is not immediately evident; however, functional insights may be gained by examining the relationship between the NR architecture and that of the nuclear basket. Just like how at least a double NR is required for a stable basket and how basket struts emanate from the double NR, the cytoplasmic filaments also emanate in a bit similar manner from the mNPC’s double CR (Figure 1B). This observation indicates the importance of higher stoichiometries of outer rings in supporting the stable structure of additional NPC modules like the basket and the cytoplasmic filaments. The additional density in the mNPC’s double CR (highlighted in Figure 1) likely represents the metazoan-specific arrangement of Nup358.^60–62^

It should be noted that the term “basket,” as traditionally defined and understood, refers to a stable architecture with struts. However, as we have shown and is consistent with observations here, the basket can be highly dynamic, can also interact with many other proteins, and indeed may have additional components, such as yPml39/mZC3HC1.^54,63^ Various studies have established that much of the pool of nuclear basket components is dynamically associated with the yNPC^39^; and, similar to yeast Nup1, Nup60, and Nup2,^40,41^ mammalian Nup153 and Nup50 are also dynamically associated with their respective NPCs.^64^ However, the major strut component in yeast (Mlp1/2) is dynamically associated,^40,41^ whereas that in mammals (Tpr) seems more stably associated with the NPC, in agreement with the near ubiquitous appearance of the basket in mNPCs (above).^12,13,65^ Moreover, we found that every double NR yNPC had a clearly associated basket, whereas the single NR NPCs did not have clear morphologically discernible baskets. However, in seeming contradiction, most yNPCs associate with basket components *in vivo*.^6,23–25,39,66^ A reconciliation of this apparent contradiction can be made simply, as follows: we know that the majority of the yeast Mlp1/2 pool is also dynamic (above); therefore, we suggest that for the single NR yNPCs, their association with basket components, including Mlp1/2, is transient, and furthermore that the basket components are more flexible and perhaps not stoichiometric,^17^ such that their presence is difficult to establish by in-cell cryo-electron microscopy (cryo-EM). By contrast, we suggest that the double NR yNPCs, by virtue of their possession of an additional set of basket protein binding sites in their extra NR, can bind much more stably to Mlp1/2, thus accounting for the ubiquitous observation of basket struts in the double NR yNPCs. This idea also agrees with the observation, using scanning electron microscopy, of complete baskets on only a subset of yNPCs, although all had some strut-like nuclear filaments.^17^ We may also propose a function as to why yeast possess these minority double NR yNPC forms; while most yNPCs (with single NRs) utilize a rapidly reversible recruitment mechanism for the basket during mRNA export,^39,66^ as masters of “bet hedging”^67^ yeast keep in reserve an NPC subset with pre-assembled basket, perhaps to accommodate rapid changes in mRNA export or to ensure maintenance of epigenetic memory.^68^ In mNPCs, the presence of a double NR on essentially all NPCs ensures the stable association of Tpr and a morphologically recognizable basket.

When an NPC acquires a double NR, it not only becomes more stably associated with Mlp1/2; it also apparently becomes capable of more stably binding yNup1, yNup60, and yNup2, as the presence of the extra ring provides observed additional binding sites for these proteins on the NPC (Figure 5A; Video S1). The binding of these proteins to this aforementioned hub in the NE has been shown to be managed by posttranslational modifications.^69,70^ Interestingly, a similar phosphorylation-driven mode of assembly and disassembly is found in flexible connector-containing Nups of cells that undergo NE breakdown, as we previously suggested.^5^ Our cryo-ET analysis was not able to resolve a basket in the double NR-containing *T. gondii* NPCs, most likely due to the limited dataset, and thus we cannot at this stage rule out the presence of a highly divergent basket structure or loss of the basket, as we were unable to identify obvious Tpr homologs in this organism. However, this may also suggest that the presence of a double NR might not be sufficient to sustain a highly stable basket assembly. Indeed, even in yeast and vertebrates, the presence of a double NR does not guarantee the continued presence of a full stable basket, as it has been shown that under stress^71,72^ or under certain cellular states,^69,70,73^ the nuclear basket can dissociate from the NPC.

It was proposed that the structural resilience of the NPC is achieved via an architecture that combines flexible and rigid modules, akin to the design of a suspension bridge.^5,6^ For the basket, we define a flexible module as one that changes its structure between yeast and mouse models, regardless of its position and orientation relative to the rest of the NPC. By contrast, we define a rigid module as one that does not change its structure between the yeast and mouse models but may or may not change its position relative to the NPC. According to these definitions, the flexible modules include the unstructured domains of the NR anchor module (violet, Figures 5C and 5D; Video S1) and the basket distal module (green, Figures 4B and 5D). By contrast, the rigid modules include the subunits of the double NR (pink and tan, Figures 5C and 5D; Video S1), structured domains of the NR anchor module, and the basket strut module (green, Figures 5C and 5D; Video S1). However, it is not clear whether the flexible modules are dynamic or simply reflect static differences between yeast and mouse. Assuming the former, we can explain the resilience of the basket architecture as follows: The N-terminal amphipathic helices (Figure S4C) within the yNup60/mNup153 of the NR anchor module may serve as critical anchors to the NE (gray, Figure 4), in full agreement with previous findings.^26,53^ The flexible regions of the NR anchor module connect to the rigid double NR and basket strut module, mimicking the role of suspension cables that connect rigid columns and the roadway of a suspension bridge.^5,6,53^ The suspension bridge-like architecture may provide the necessary resilience of the basket while transporting large cargoes.

It was previously observed that the C terminus of Mlp1 acts as a necessary transient docking site for messenger ribonucleoproteins (mRNPs) during mRNA export^57^ and that the nucleocytoplasmic transport of large molecules through the NPC involves an increase in the basket ring radius.^20^ Our model rationalizes these observations as follows: the C terminus of Mlps is located in the basket distal density. If the difference between the yeast and mammalian models was indicative of the basket structural dynamics, the dynamics of the basket would involve the movements of the basket subunit and conformational changes of anchor Nups (Figures 5E and 5F; Video S1)^24,74^; it remains to be determined whether or not the differences between the two species models reflect different basket dynamics. Moreover, the flexible linkers connecting the coiled-coil segments from the yMlps/mTprs dimer in the struts may also afford the flexibility to the basket to contract and expand as seen for other rings.^5,28,44^ Such motions could account for the expansion and contraction of the basket seen during passage of large cargoes.^20^ A significant portion of the basket distal density in yeast remains unaccounted for in our integrative model, possibly indicating the presence of cargo, transport factors, or other elements, which aligns with the observation that this density could be a docking site for cargo (Figures 5E and 5F; Video S1). Unlike the yNPC, the basket distal density in the mNPC is considerably smaller, and thus, a larger proportion of its density is accounted for in its models (Figures 5E and 5F; Video S1).

The sizes of many mRNPs range from ~200 to 600 Å, smaller than the diameters of the rings, including that of the basket, potentially allowing them to pass unaltered across the NPC.^75,76^ However, some mRNPs can have elongated shapes or be much larger than the basket’s diameter^20,75,76^; in these cases, their shape can be adjusted or remodeled to pass through the NPC, and the basket too can remodel as seen during passage of such large cargoes.^20,77^ These adjustments or remodelings could commence while the mRNP is docked at the basket distal density.

Our data show that the basket-forming proteins are surrounded by an exclusion zone of ~20 nm, but indicate that there are no disordered domains stemming from the basket in this zone. This observation will inform future targeted studies into the molecular basis of this exclusion, e.g., having a role in restraining chromatin.^21^ Apart from chromatin exclusion, NPCs also influence chromatin organization through the context-specific localization of either active or repressed genes to the NPC.^78–81^ These localizations require basket Nups such as yNup1, yNup2, yNup60, mNup153, and Mlps, implying the basket’s critical role in these localizations.^78–81^ Similar to mRNA docking, these localizations may also occur at the basket distal density, consistent with reported interactions between the C termini of Mlps (which we show to form the distal density) and complexes involved in genes localization to the NPC.^82^ Interestingly, chromatin organization is not only impacted by the presence of the basket but also by its systematic absence, as shown recently that basketless yNPCs are involved in a process of subtelomeric gene silencing.^83^

In the future, identifying different macromolecules docked onto the basket distal density using template-matching and deep-learning approaches to identify cargo in in-cell tomograms, complemented with cross-linking mass spectrometry data to determine molecular interactions, would expand our understanding of the role of the basket distal density. For example, in *Chlamydomonas reinhardtii*, proteasomes were found associated with the NPC ~550–600 Å from the NPC’s NR,^84^ roughly the same length as the struts of the basket (ending onto distal density) observed in this study. This observation not only solidifies the model positing the basket distal density as the docking site for various macromolecules but also expands the role of the NPC in locally harboring proteasomes, which may play important roles in ensuring correct folding of imported proteins and may coordinate with NPC-associated SUMOylation (SUMO; small ubiquitin-like modifier) and ubiquitylation pathways for various regulatory processes, including DNA damage repair.^85,86^ Indeed, though the nuclear basket is known to play functional roles in gene regulation, chromatin organization, mRNA surveillance, and mRNA processing before nuclear export, the exact mechanisms by which it facilitates and regulates these processes remain poorly understood. Moreover, the basket has been reported to dilate during nuclear export of large mRNPs, potentially connecting conformation changes of the basket with mRNP remodeling and export.^20^ Another set of interesting future investigations could include further characterization of the variability of the NPC’s outer rings across different species and, more importantly, the reason for this variability. One possible reason is the stabilization of additional modules like the basket, which our analysis suggests to be stabilized only with at least a double NR, implying that there are populations of NPCs with stably associated baskets and ones to which basket components are more dynamically associated or transiently recruited.^39^ Our work underscores that the nuclear basket can exhibit significant structural variation between NPCs within a single cell, even to the point of being entirely absent, but the functional reasons for this variability are as yet only incompletely understood. Furthermore, the role of the basket has primarily been discussed in the context of nuclear export, not import. However, it would be interesting to explore what happens to the basket during import, including for large cargoes like HIV capsids that can pass through the NPC as intact entities,^87,88^ but in doing so can disrupt the NPC’s architecture.^88^ Do imported cargoes get momentarily docked on the basket distal density for specialized processing? What kind of processing occurs? We hope that the structures revealed in this manuscript will provide a groundwork map to inspire studies into these and other open questions concerning the many and varied functions of the nuclear basket.

### Limitations of the study

While the current basket models provide insights into their overall architectures, the current resolution of the yeast and mouse cryo-ET maps hinders the construction of residue-level structural models and restricts our ability to interpret the structural differences between yeast and mouse. Although extensive biochemical data were used to model the yeast basket, the mouse basket model relied on cryo-ET maps and inferred structural similarity to the yeast basket. Consequently, without additional orthogonal data to characterize the mouse basket, we cannot unequivocally determine whether the observed differences between the yeast and mouse basket models reflect species-specific variations or distinct functional states. Furthermore, key nuclear basket components, such as yPml39/mZC3HC1, have not been included in the model due to insufficient data to define their precise positions within the structure. We were unable to locate and visualize the nucleolus in the in-cell tomograms of yeast, which may have allowed us to better interpret the cellular environment of the different yNPCs (single NR without a stable basket and double NR with basket). This interpretation would be pertinent because fluorescence microscopy shows that yNPCs adjacent to the nucleolus are mostly depleted of basket proteins like Mlps,^5,23,39,89^ underscoring a potential difference in NPC composition near the nucleolus versus the rest of the nucleus.

## STAR★METHODS

### RESOURCE AVAILABILITY

#### Lead contact

Further information and requests for resources and reagents should be directed to and will be fulfilled by the lead contact, Elizabeth Villa (evilla@ucsd.edu).

#### Materials availability

Strains used in this study will be distributed without restriction upon request.

#### Data and code availability

- Cryo-ET maps have been deposited in the EMDB with the following accession codes: Yeast NPC (EMD-44377, EMD-44372, EMD-45255, EMD-45197, EMD-45198, EMD-45256, EMD-45199, EMD-45200, EMD-45201, EMD-45202, EMD-45203, EMD-45204, EMD-45205), Mammalian NPC (EMD-44379, EMD-45257, EMD-45216, EMD-45258, EMD-45219, EMD-45220, EMD-45222, EMD-45223, EMD-45227), Protozoan NPC (EMD-44381, EMD-45259, EMD-45228, EMD-45260, EMD-45229, EMD-45230, EMD-45231, EMD-45232, EMD-45233). Integrative models have been deposited in the PDB-Dev with the following codes: Collection of all models (PDBDEV: PDBDEV_G_1000004), Yeast NPC (PDBDEV: PDBDEV_00000386, PDBDEV: PDBDEV_00000387), and Mammalian NPC (PDBDEV: PDBDEV_00000384, PDBDEV: PDBDEV_00000385). Cross-linking data have been deposited at Zenodo with the 10892434 accession code (https://zenodo.org/).
- Software scripts and data for integrative modeling are available at: https://github.com/integrativemodeling/NPC_Basket and archived at Zenodo with accession code 12561838.
- Any additional information required to reanalyze the data reported in this paper can be requested from the lead contact, Elizabeth Villa (evilla@ucsd.edu).

### EXPERIMENTAL MODEL AND STUDY PARTICIPANT DETAILS

Many cell lines and yeast strains used in this study are standard cell lines and strains, which are readily available via multiple sources. These strains/cell lines plus more specialized ones used in the study will also be distributed without restriction upon request to the lead contact, Elizabeth Villa (evilla@ucsd.edu).

### METHOD DETAILS

#### Cell culture, vitrification and sample preparation

W303 yeast cells were cultured in yeast extract peptone dextrose (YPD) media supplemented with adenine hemisulfate. These cells in the log-growth phase were collected and deposited on glow-discharged Quantifoil grids (R 2/1, Cu 200-mesh grid, Electron Microscopy Sciences), as described previously.^5^ The mouse fibroblasts cells (NIH3T3) were cultured at 37°C and 5% CO2 in Dulbecco’s Modified Eagle Medium (DMEM) with 10% fetal calf serum. Cells were seeded onto glow-discharged and Fibronectin-coated Quantifoil grids (R1/4, Au 200-mesh grid, Electron Microscopy Sciences). Following this seeding, the cells were cultured for 2 more hours on the grids to allow for their stable adherence onto the grid. In some cases, grids were micropatterned with 40 μm circles and treated with 100 nM jasplakinolide for a further two hours after seeding. The tachyzoites (*T. gondii* in rapid growth phase) were thawed out from liquid nitrogen and cultivated in human foreskin fibroblasts (HFFs) using Dulbecco’s modified Eagle’s medium (DMEM), with medium changes every 12 to 24 hours. To collect tachyzoites, trypsin-treated, parasite-infected HFFs were mechanically disrupted using a 27-gauge syringe, and the mixture was filtered to separate tachyzoites from HFF debris. The tachyzoites were then centrifuged, resuspended in DMEM with 30% fetal bovine serum (FBS) and 10% DMSO, and deposited on EM grids for vitrification, as described previously.^107^ Excess media was manually blotted from the back (opposite to the carbon film and seeded cells). Grids were plunge-frozen in a liquid ethane-propane mixture (50/50 volume, Airgas) using a custom-built vitrification device (Max Planck Institute for Biochemistry, Munich). Frozen grids were clipped into AutoGrids with a milling slot (Thermo Fisher Scientific) to allow milling at shallow grazing angles as described previously.^32,108^ Cryo-FIB milling was performed in an Aquilos Dual-Beam (Thermo Fisher Scientific) as described previously.^32,108^

#### Tilt series acquisition

Tilt series were acquired on the Titan Krios G3 (Thermo Fisher Scientific) at 300 keV with either a K2 detector and Quantum 968 LS post-column energy filter or a K3 Summit detector with 1067HD BioContinuum post-column energy filter in counting and dose fractionation modes (Gatan). The tilt-series parameters were as follows: tilt range: ± 45–60°, pixel size of 3.45 Å (yeast), 1.32 Å (mouse fibroblasts), 3.328 Å (*T. gondii*), tilt increment: 3° (higher for some samples), effective defocus range: −2 to −11 μm, total fluence: ~100–180 e-/Å 2. All image acquisition was done using SerialEM software.^93,109^ For some tilt-series, parallel cryo-electron tomography (PACE-tomo) scripts were used.^94^ In total, 1449, 136, and 19 (total: 1604) tilt series were used for yeast, mouse, and *T. gondii*, respectively. This data set included 153 tilt-series of yeast from EMPIAR-10466.^8^

#### Subtomogram analysis

Frames of the tilt images were motion-corrected using whole-frame motion and organized into stacks in WARP.^95,110^ The motion-corrected tilt series were then aligned in AreTomo.^102^ The aligned tilt-series stacks were subsequently re-imported into WARP for CTF estimation, defocus handedness determination, and final reconstruction.^95,110^ The CTF estimation and defocus handedness were manually inspected and further refined as needed. In tomograms, nuclear pores were manually picked in IMOD.^101^ For each pore, in addition to the coordinate of the pore’s center, an additional point approximately 50–100 nm on the cytoplasmic side was marked. The pores were oriented using these two points with the Dynamo dipole picking mode.^104^ The subtomograms of the pores, with these initial orientations, were generated in WARP at a pixel size of 10 Å. The total number of pores picked were ~5160 for yeast, ~220 for mouse, and ~50 for *T. gondii*, respectively. A small number of pore particles were used to generate a C8 symmetrized initial model in Relion.^96^ This initial model served as a reference for refining all the pore particles with C8 symmetry. The refinements were performed with local searches around the initial orientation (initial Euler angles), using the *sigma_ang/rot/tilt/psi* parameters to restrict the angular searches and prevent the pore particles from flipping. The term *sigma_ang/rot/tilt/psi* in Relion specifies the width of the Gaussian prior on the starting Euler angles. 3D classification (without alignments, simply referred to as classification), using C8 symmetry, was performed using a mask focused on the inner ring of the NPC to select good particles and discard bad ones. The selected NPC particles were refined further with C8 symmetry. For yeast, classification was performed using a mask focused on the nuclear ring to classify out NPCs with single and double NR, which accounted for ~77% and the remaining ~23% of total NPCs, respectively. The symmetry expansion was carried out to isolate subunits of the NPCs. These subtomograms of the subunits were then reconstructed at a pixel size of 10 Å in WARP. The *relion_reconstruct* was used to generate an average of these subtomograms for use as reference in the refinement of these subunits using a mask focused on the IR subunit. Following refinement, classification was performed, using the mask focused on the IR subunit, to select good subunits and discard bad ones. The refinements of the good subunits of IR, CR, NR, and the basket (as applicable) were then performed using their respective shape masks. All the refinements and classification of subunits were done without the use of symmetry except for the map shown in Figures 2A and S3C. The total number of subunits used in the final refinements were ~28600 for yeast (out of which, ~6600 were from the NPC with double NR), ~800 for mouse, and ~265 for *T. gondii*, respectively. The maps shown in Figures 2A and S3C, were determined by the averaging of the whole NPC particle (containing multiple subunits) using the C8-symmetrization. For this averaging, a soft-mask covering the relevant portion of the NPC in the particle, was used for alignment and averaging. This mask did not include the surrounding densities shown in the maps in Figures 2A and S3C. After the iterative alignment and averaging, a new-reconstruction, at a much bigger box size to encompass a large area containing surrounding densities was reconstructed using *relion_reconstruct* and C8 symmetrized. The 0.143-cut-off criterion of the Fourier Shell correlations (FSC) between masked and independently refined half-maps was used to estimate all the reported resolutions.^111^ The maps of the subunits of these different rings were composited to generate the final map of the entire subunit of the NPC. This composite map was fit into the map of the whole NPC (of C8 symmetry) using Chimera’s *fit-to-map* tool, and then C8 symmetrized using *relion_image_handler*. The entire processing of the data from separate organisms was done completely separately and independently. The schematic of the entire workflow and resolution estimates is also shown in Figure S1. v3.1.1 of Relion was used for all steps involving Relion.^96^ v1.09 or v1.1.0-beta1 of WARP was used for all steps involving WARP.^95,110^

#### Pairwise distances amongst yNPCs and their radial distribution function [g(r)]

The coordinates of yNPCs with single or double NR in their tomograms were obtained following their subtomogram analysis. For each tomogram, pairwise distances among all yNPCs, as well as those with single and double NR, were calculated using these coordinates. These distances were then used to estimate the g(r) for each tomogram. The g(r) values from all the tomograms were averaged to generate the final g(r) shown in Figure S2B. It should be noted that these pairwise distances and their corresponding g(r) values are averages for all yNPCs and might not apply to small subsets of yNPCs. For instance, yNPCs near the nucleolus are likely to be less enriched in double NRs (with a stable basket). This observation comes from fluorescence imaging, which has shown that yNPCs near the nucleolus lack yMlps (one of the basket-Nups) and have a low level of NR-Nups, indicating a preference for single NR without the basket.^5,23,39,89^

#### Chemical cross-linking and MS (CX-MS) analysis of affinity-purified yeast NPCs

CX-MS of Mlp1-PPX-PrA tagged, affinity purified, native, whole NPCs have been described in detail in Akey et al.^5^ and Kim et al.^6^ To expand and complement these datasets with cross-links mapping exclusively to basket Nups fully assembled into the NPC, we used NPCs affinity purified using Dbp5-PPX-GFP and Gle1-PPX-PrA as the handles using a similar protocol, with the following modifications: After native elution, 1.0 mM disuccinimidyl suberate (DSS) was added and the sample was incubated at 25°C for 40 minutes with shaking (1,200 rpm). The reaction was quenched by adding a final concentration of 50mM freshly prepared ammonium bicarbonate and incubating for 20 minutes with shaking (1,200 rpm) at 25°C. Crosslinked NPCs were pelleted by spinning for 20 minutes in a TLA-55 rotor (Beckman) at 25,000 rpm. The pelleted samples (~50 mg) were resuspended in 1xLDS with 25 mM DTT and incubated at 70°C for 10 minutes. Reduced samples were alkylated by adding a final concentration of 100 mM iodoacetamide and incubating in the dark at 25°C for 30 minutes, followed by addition of an additional 25 mM DTT and further incubation for 15 minutes. Alkylated and reduced samples were denatured at 98°C for 10 minutes and then loaded into 4% SDS-PAGE Bis-Tris gel and run for 10 minutes at a constant 120 V to reduce the complexity of the sample. For in-gel digestion, the high-molecular-weight-region gel bands corresponding to cross-linked NPC proteins were sliced and proteolyzed by trypsin as previously described.^6^ In brief, gel plugs were crushed into small pieces and 5–10μg of sequencing-grade trypsin (Promega) per ~100 μg protein were added. Trypsin was supplied in two equal additions and incubated with gel pieces at 37°C in 50 mM ammonium bicarbonate, 0.1% (w/v) Rapigest (Waters). After the first addition, the samples were incubated for 4 hours. After the second addition, the samples were incubated overnight. Peptides were extracted by formic acid and acetonitrile, and dried partially by vacuum centrifugation. To remove the hydrolytic insoluble by-products of Rapigest, the sample was centrifuged at 20,000g for 10 min. The solution was transferred to another tube and then further dried by vacuum centrifugation. Peptides were separated into 6–7 fractions by high pH reverse phase fractionation in a pipet tip self-packed with C18 resin (ReproSil-Pur 120 AQ, 3μm, Dr. Maisch GmbH). Each peptide fraction was resuspended in 5% (v/v) methanol, 0.2% (v/v) formic acid and loaded onto an EASY-Spray column (Thermo Fisher Scientific, ES800, 15cm × 75mm ID, PepMap C18, 3mm) via an EASY-nLC 1200 (Thermo Fisher Scientific). The column temperature was set to 35°C. Using a flow rate of 300 nl/min, peptides were gradient-eluted (3–6% B, 0–6 min; 6–34% B, 6–97 min), where mobile phase B was 0.1% (v/v) formic acid, 95% (v/v) acetonitrile and mobile phase A was 0.1% (v/v) formic acid in water. An Orbitrap Fusion Lumos Tribrid (Thermo Fisher Scientific) was used to perform online mass spectrometric analyses. Full MS scans were performed at least every 5 s. As time between full scans allowed, ions with charge states +4 to +8 were fragmented by higher-energy collisional dissociation in descending intensity order with a maximum injection time of 800 msec. Both precursors and fragments were detected in the Orbitrap. The raw data were searched with pLink^105^ and pLink2^106^ with cysteine carbamidomethyl as a fixed modification and methionine oxidation as a variable modification. The initial search results were obtained using a default 5% false discovery rate (FDR) expected by the target-decoy search strategy. Spectra corresponding to basket components were selected and manually verified to ensure data quality.^6^

#### Integrative modeling of the basket

Coarse-grained structural models of the yeast and mouse baskets were computed using an integrative modeling approach,^6,48–52^ based on information from varied experiments, physical principles, statistical preferences, and prior models (Table S1). The yBasket model includes the yMlp1/2, FG Nups (yNup1, yNup2, and yNup60), as well as the double NR Nups (yNup120, yNup85, yNup145c, ySec13, ySeh1, yNup84, and yNup133).^4,24–26^ The mBasket model includes the orthologs of yeast Nups (mTpr, mNup50, mNup153, mNup160, mNup85, mNup96, mSec13, mSeh1, mNup107, mNup133, mNup43, and mNup37).^12,13^ Modeling positioned the yMlp/mTpr and FG Nups relative to the fixed double nuclear ring; in addition, it optimized the conformations of the disordered Nup regions. The modeling protocol was scripted using the Python Modeling Interface (PMI) package version a41075a, which is a library for modeling macromolecular complex structures based on our open-source *Integrative Modeling Platform* (IMP) package version 2.19 (https://integrativemodeling.org).^50^

##### Stage 1: Gathering information

The sequences of the basket Nups were obtained from the Uniprot database^112^ (Tables S1 and S2). Their stoichiometry in the yNPC was previously determined by quantitative mass spectrometry of the isolated yNPC complex^6^ (Table S2). In total, 626 unique intra- and intermolecular DSS cross-links were previously identified using mass spectrometry.^6,24,113^ The cryo-ET map described here informed the overall shape of the basket and its anchoring on the double nuclear ring. The structural model of the yNup84 complex of the double NR was previously determined by an integrative approach.^5^ The structural model of the yMlps was informed by the coiled-coil propensities and heptad repeat alignments and was generated using COCONUT software^91^ (Figures 3, stage 2, S4A, and S4B; Table S1). The yNup2 structural model was obtained from the AlphagFold database version 4 (Table S2). Direct physical interactions between yNup60, yNup2, and yMlp1 were determined by *in vitro* binding assays^53^ (Table S1). Previously determined immuno-electron microscopy images help localize the terminal domains of the yMlps.^24^ Similar information was used for mBasket modeling^12,114^ (Figures 3 and S4; Tables S1 and S3).

##### Stage 2: Basket representation and spatial restraints

###### Basket representation.

We modeled only a single subunit out of the eight subunits comprising the entire yBasket, mostly without explicitly considering the interfaces between the eight symmetry units in the yNPC. This simplification was possible because the explored positions and conformations of the basket components do not clash with each other across the symmetry unit interfaces, courtesy of their anchoring on the fixed double NR. The stoichiometries of yMlp1 and yMlp2 are ambiguous.^6^ Thus, we used two copies of poly-alanine per symmetry unit (yMlp), representing both yMlp1 and yMlp2; the yMlp length was set to that of yMlp1. The model also included a single copy of yNup1 and two copies of yNup2, yNup60, and the heptameric yNup84 complex. FG repeats were not included in the model. In total, the yBasket model consists of 21 protein subunits of 12 types (Table S2). A similar representation was used for mBasket modeling with two copies of mTpr, two copies of mNup50, mNup153, and the nonameric mNup107 complex (Tables S1 and S3).^36,115,116^ Thus, the mBasket model consists of 24 protein subunits of 12 types (Table S3). Each component was represented in a multiscale fashion to balance the accuracy of the formulation of restraints and the efficiency of structural sampling (Figure S4C; Tables S2 and S3).

###### Spatial restraints on yeast and mouse baskets.

The subset of input information was converted to spatial restraints for scoring alternative models.^50^ These restraints include upper bounds on pairs of crosslinked residues based on chemical crosslinks, the correlation coefficient between Gaussian Mixture Models of a model and the cryo-ET map, positional restraints on NTD/CTD domains of yMlps based on immuno-EM localizations, distance restraints between pairs of domains based on affinity co-purification data, positional restraints on residue segments predicted to lie within the nuclear envelope, connectivity restraints between consecutive pairs of beads in a subunit, excluded volume restraints between non-bonded pairs of beads (Table S2).^50^ For the mBasket (Table S3), crosslinking and affinity copurification data were unavailable. However, we supplemented the remaining mBasket restraints with structural equivalence restraints; these distance restraints are designed to maximize the similarity between the mouse and yeast models across the aligned residues, subject to the satisfaction of the remaining restraints.

##### Stage 3: Structural sampling

The initial positions and orientations of rigid bodies and flexible beads were randomized except for the double NR rigid body (Table S2), whose position was obtained by fitting into the cryo-ET map, ensuring accurate alignment with experimental cryo-ET maps (Figure 3 Stage 3; Table S2). Structural sampling of rigid body positions and orientations as well as flexible bead positions, was performed using the Replica Exchange Gibbs Monte Carlo (MC) algorithm (Table S2).^117,118^ Each MC step consisted of a series of random transformations (i.e., rotations and translations) applied to the rigid bodies and flexible beads. The same sampling protocol was used for mBasket modeling, except that the starting structure mimicked the yBasket model (Table S3). Thus, by construction, any potential differences between the yeast and mouse basket models are a direct consequence of the differences on the cryo-ET maps and other input information.

##### Stage 4: Analysis and validation

Model validation followed four steps^52,119^: (i) selection of the models for validation, (ii) estimation of sampling precision (Figure S5A), (iii) estimation of model precision, and (iv) quantification of the degree to which a model satisfies the information used and not used to compute it (Tables S2 and S3; Figures S5 and S6).^52,120^ Integrative modeling iterated through the four stages to find a set of models that satisfy our validation criteria listed above. In each iteration, we considered the input information, representation, scoring, and sampling guided by an analysis of models computed in the preceding iteration of the modeling. For example, the initial low precision of the yBasket model encouraged us to improve the resolution of the cryo-ET map by averaging a larger number of subtomograms; and the initial inability to find yBasket models that satisfied both cryo-ET and crosslinking data encouraged us to increase the resolution and flexibility of the coiled-coil representations, re-defining the coiled-coil segments,^97^ disorder predictions for FG Nups,^112^ and adding proximity restraints to some components.

#### Figures

All figures depicting cryo-ET maps and models were generated using Chimera/ChimeraX^99,100^ and its RMSF plugin (https://github.com/salilab/rmf_chimerax).

### QUANTIFICATION AND STATISTICAL ANALYSIS

Resolution of all cryo-ET maps were estimated using FSC-0.143 criterion in Relion.^96,111^ Local resolution maps were calculated in EMAN2.^121^ These details of quantification and all statistical analyses have also been described in the relevant sections of the method details.

## Supplementary Material

MMC1

MMC2

3

## Figures and Tables

**Table:** KEY RESOURCES TABLE

REAGENT or RESOURCE	SOURCE	IDENTIFIER
Antibodies		

Rabbit IgG Protein A Purified	Innovative Research	Cat.# IR-RB-GF; RRID:AB_1501660

Chemicals, peptides, and recombinant proteins		

PreScission protease	GE Healthcare Life Sciences	Cat.# 27–0843-01
GelCode Blue Stain Reagent	Thermo Fisher Scientific	Cat.# 24592
DSS(DiSuccinimidylSuberate)-H12	Creative molecules	Cat.# 001S
Trypsin Sequencing Grade, modified	Thermo Fisher Scientific	Cat.# 25200072
Iodoacetamide	Sigma	Cat.# I6125–10 g
Nupage LDS Sample buffer	Thermo Fisher Scientific	Cat.# NP0007
Fibronectin	Sigma-Aldrich, Inc.	Cat.# 341631
Dulbecco’s modified Eagle’s medium	Thermo Fisher Scientific	Cat# 11995073
Antibiotic-Antimycotic	Thermo Fisher Scientific	Cat# 15240062
Calf serum	Colorado Serum Company	Cat# 31332
Fetal Bovine Serum	Thermo Fisher Scientific	Cat# A3840002
Jasplakinolide	Thermo Fisher Scientific	Cat# J7473
50% Ethane Balance Propane	Airgas	Cat# X02PR50C33A0000

Critical commercial assays		

Dynabeads M270 Epoxy	Thermo Fisher Scientific	Cat # 143.02D

Deposited data		

Yeast NPC: Full C8 NPC’s composite of double NR	This paper	EMD-44377
Yeast NPC: Full C8 NPC’s composite of single NR	This paper	EMD-44372
Yeast NPC: Full C8 NPC’s consensus	This paper	EMD-45255
Yeast NPC: C1 asymmetric subunit’s composite of double NR	This paper	EMD-45197
Yeast NPC: C1 asymmetric subunit’s composite of single NR	This paper	EMD-45198
Yeast NPC: C1 asymmetric subunit’s consensus	This paper	EMD-45256
Yeast NPC: Focused CR’s subunit	This paper	EMD-45199
Yeast NPC: Focused IR’s subunit	This paper	EMD-45200
Yeast NPC: Focused NR’s subunit of single NR	This paper	EMD-45201
Yeast NPC: Focused NR’s subunit of double NR	This paper	EMD-45202
Yeast NPC: Focused basket’s subunit	This paper	EMD-45203
Yeast NPC: Focused membrane’s subunit (single NR)	This paper	EMD-45204
Yeast NPC: Focused membrane’s subunit (double NR)	This paper	EMD-45205
Mammalian NPC: Full C8 NPC’s composite	This paper	EMD-44379
Mammalian NPC: Full C8 NPC’s consensus	This paper	EMD-45257
Mammalian NPC: C1 asymmetric subunit’s composite	This paper	EMD-45216
Mammalian NPC: C1 asymmetric subunit’s consensus	This paper	EMD-45258
Mammalian NPC: Focused CR’s subunit	This paper	EMD-45219
Mammalian NPC: Focused IR’s subunit	This paper	EMD-45220
Mammalian NPC: Focused NR’s subunit	This paper	EMD-45222
Mammalian NPC: Focused basket’s subunit	This paper	EMD-45223
Mammalian NPC: Focused membrane’s subunit	This paper	EMD-45227
Protozoan NPC: Full C8 NPC’s composite	This paper	EMD-44381
Protozoan NPC: Full C8 NPC’s consensus	This paper	EMD-45259
Protozoan NPC: C1 asymmetric subunit’s composite	This paper	EMD-45228
Protozoan NPC: C1 asymmetric subunit’s consensus	This paper	EMD-45260
Protozoan NPC: Focused CR’s subunit	This paper	EMD-45229
Protozoan NPC: Focused IR’s subunit	This paper	EMD-45230
Protozoan NPC: Focused NR’s subunit	This paper	EMD-45231
Protozoan NPC: Focused membrane’s subunit	This paper	EMD-45232
Protozoan NPC: Focused lumenal ring’s subunit	This paper	EMD-45233
Single collection of all (yeast + mammalian) basket models	This paper	PDBDEV: PDBDEV_G_1000004
Yeast NPC: C1 asymmetric subunit basket model	This paper	PDBDEV: PDBDEV_00000386
Yeast NPC: Full C8 basket model	This paper	PDBDEV: PDBDEV_00000387
Mammalian NPC: C1 asymmetric subunit basket model	This paper	PDBDEV: PDBDEV_00000384
Mammalian NPC: Full C8 basket model	This paper	PDBDEV: PDBDEV_00000385
Yeast NPC: Chemical Cross-linking with Mass Spectrometry readout datasets	This paper; Akey et al.^5^; Kim et al.^6^	Zenodo: 10892434
Protozoan NPC: Some Tilt-series of *S. cerevisiae* NPC	Allegretti et al.^8^	EMPIAR-104661
Yeast NPC modeling: yNup84 complex dimer	Akey et al.^5^	PDB: 7N84
Yeast NPC modeling: Fitted yNup84 complex dimer on input map	This paper	Zenodo: 12561838
Yeast NPC modeling: Derived from yNup2 AlphaFold model	This paper; Jumper et al.^90^	Zenodo: 12561838
Yeast NPC modeling: Generic Mlps coiled-coil segments models	This paper; Soni and Madhusudhan^91^	Zenodo: 12561838
Yeast NPC modeling: Files with input data, scripts, and output resultsfor the integrative modeling of yBasket	This paper	Zenodo: 12561838 and https://github.com/integrativemodeling/NPC_Basket
Mammalian NPC modeling: mNup107 complex dimer model	This paper	Zenodo: 12561838
Mammalian NPC modeling: Fitted mNup107 complex dimer on input map	This paper	Zenodo: 12561838
Mammalian NPC modeling: Derived from mNup50 AlphaFold model	This paper; Jumper et al.^90^	Zenodo: 12561838
Mammalian NPC modeling: Tprs coiled-coil segments models	This paper; Soni and Madhusudhan^91^	Zenodo: 12561838
Mammalian NPC modeling: Files with input data, scripts, and output resultsfor the integrative modeling of mBasket	This paper	Zenodo: 12561838 and https://github.com/integrativemodeling/NPC_Basket

Experimental models: Cell lines		

NIH3T3 Cell line	ATCC	CRL-1658
*Toxoplasma gondii* RH strain	Readily available strain	*Toxoplasma gondii RH strain*
Human foreskin fibroblasts	Readily available strain	Human foreskin fibroblasts (HFF)

Experimental models: Organisms/strains		

MATa ade2–1 ura3–1 his3–11,15 trp1–1 leu2–3,112 can1–100	Kim et al.^6^	W303
MATa ade2–1 ura3–1 his3–11,15 trp1–1 leu2–3,112 can1–100 MLP1-PPX-ProteinA::HIS5	Kim et al.^6^	Mlp1-PPX-PrA
MATa his3Δ200 trp1Δ63 leu2Δ0 met15Δ0 ura3Δ0	Brachmann et al.^92^	BY4733
MATa his3Δ200 trp1Δ63 leu2Δ0 met15Δ0 ura3Δ0 Dbp5-PPX-GFP::TRP1	This paper	Dbp5-PPX-GFP
MATα ade2–1 ura3–1 his3–11,15 trp1–1 leu2–3,112 can1–100 Gle1-PPX-ProteinA::HIS5	This paper	Gle1-PPX-PrA

Software and algorithms		

SerialEM	Mastronarde^93^	https://bio3d.colorado.edu/SerialEM/
PACE-Tomo	Eisenstein et al.^94^	https://github.com/eisfabian/PACEtomo
WARP	Tegunov and Cramer^95^	http://www.warpem.com/warp/
Relion 3.1	Nakane et al.^96^	https://github.com/3dem/relion
IMP, version 2.19	Russel et al.^50^	https://integrativemodeling.org/
PCOILS	McDonnell et al.^97^; Gabler et al.^98^	https://toolkit.tuebingen.mpg.de/tools/pcoils
chimera/Chimerax	Pettersen et al.^99^; Pettersen et al.^100^	https://www.cgl.ucsf.edu/chimerax/
COCONUT, version 1.0	Soni and Madhusudhan^91^	https://github.com/neeleshsoni21/coconut
IMOD Package	Kremer et al.^101^	https://bio3d.colorado.edu/imod/
AreTomo	Zheng et al.^102^	https://msg.ucsf.edu/software
EMAN2	Chen et al.^103^	https://blake.bcm.edu/emanwiki/EMAN2
AlphaFold2	Jumper et al.^90^	https://github.com/google-deepmind/alphafold
Dynamo	Castaño-Díez et al.^104^	https://wiki.dynamo.biozentrum.unibas.ch/w/index.php/Main_Page
pLink and pLink2	Yang et al.^105^; Chen et al.^106^	http://pfind.ict.ac.cn/software/pLink/

Other		

Custom made plunger	Max-Planck-Institute for Biochemistry	N/A
Mass Spectrometer	Thermo Fisher Scientific	Orbitrap Fusion Lumos Tribrid
Liquid Chromatograph	Thermo Fisher Scientific	Easy-nLC 1200
Easy-Spray column	Thermo Fisher Scientific	ES800
NuPage 4–12% Bis-Tris Gel 1.0mm × 10 well	Thermo Fisher Scientific	Cat.# NP0321Box
Quantifoil R 2/1 200 Mesh, Cu	Electron Microscopy Sciences	Q2100CR1
Quantifoil R 1/4, 200 Mesh, Au	Electron Microscopy Sciences	Q210AR-14
Titan Krios G3 (300 kV Cryo-transmission electron microscope)	Thermo Fisher Scientific	Titan Krios G3.
Energy filter and direct electron detector	Gatan	K2 detector and Quantum 968 LS post-column energy filter or a K3 Summit detector with 1067HD BioContinuum post-column
Aquilos cryo-FIB Dual-Beam	Thermo Fisher Scientific	Aquilos cryo-FIB
Whatman filter paper #1	Whatman	Cat# 1001 090

## Highlights

- A stable basket is anchored by a hub of Nups into a double nuclear ring
- Mlps/Tprs form struts; their N/C termini form a distal density that docks mRNA
- A 20-nm exclusion zone around the basket suggests its role in chromatin organization
- The stoichiometry of the outer rings is variable across and within species
