# Supplementary material for: The molecular architecture of the nuclear basket
Source: Cell. Author manuscript; Available in PMC 2024 Sep 22. (PMC11416316; doi:10.1016/j.cell.2024.07.020)
Supplement: MMC1 [file NIHMS2016536-supplement-MMC1.pdf]

Cell, Volume *187*

Supplemental information

The molecular architecture of the nuclear basket

Digvijay Singh, Neelesh Soni, Joshua Hutchings, Ignacia Echeverria, Farhaz Shaikh, Madeleine Duquette, Sergey Suslov, Zhixun Li, Trevor van Eeuwen, Kelly Molloy, Yi Shi, Junjie Wang, Qiang Guo, Brian T. Chait, Javier Fernandez-Martinez, Michael P. Rout, Andrej Sali, and Elizabeth Villa

Supplemental Information for

The Molecular Architecture of the Nuclear Basket

Digvijay Singh, Neelesh Soni, Joshua Hutchings, Ignacia Echeverria, Farhaz Shaikh, Madeleine Duquette, Sergey Suslov, Zhixun Li, Trevor van Eeuwen, Kelly Molloy, Yi Shi, Junjie Wang, Qiang Guo, Brian T. Chait, Javier Fernandez-Martinez, Michael P. Rout, Andrej Sali, Elizabeth Villa

Table S1. Information and spatial restraints for Integrative modeling of yeast and mouse basket. Related to Figures 3-5 and Figures S4-S6.
For each basket model (column 1), a list of experimental techniques is shown in column 2 that provide the spatial information (column 3) for Integrative modeling. Column 4 provides a reference to the original dataset or method.

Model	Experimental Technique	Spatial Information	Reference
Yeast	Quantitative mass spectrometry and biochemical quantification	Stoichiometry of Nups (Table S2)	Kim et al., 2018[1]
	Chemical cross-linking with mass spectrometry readout	626 intra and intermolecular crosslinks among yNups and yMlps	Kim et al., 2018 and Niepel et al., 2013[1,2] & This paper
	Cryo-ET	The overall shape of the basket, including the basket distal density	This paper
	Integrative models and atomic structural models	yNup84 complex dimer model, yNup2 structural model (Table S2)	Akey et al., 2022, Jumper et al., 2021, Varadi et al. 2022[3–5]
	Bioinformatics information	Sequence information, coiled-coil propensities of the yMlps, alignments. Connectivity between residues.	McDonnell et al., 2006[6]
	Immuno-EM	Upper and lower bound on the N-/C- terminus of yMlps	Niepel et al., 2013[2]
	Biochemical experiments including *in vitro* binding assays, proximity labeling	Interactions between yNup60^{MBM} (240-318) and yMlp1 (382-620), Interaction between yNup60^{N2BM} (505-539) and yNup2 (83-136), nuclear envelope binding domain of yNup1, yNup60	Cibulka et al. 2022, and Meszaros et al. 2015[7,8]
Mouse/Human	Quantitative mass spectrometry and biochemical quantification	Stoichiometry of Nups (Table S3)	Li et al., 2021 and Ori et al., 2013[9,10]
	Cryo-ET	The overall shape of the basket, including the basket distal density	This study
	Integrative models and atomic structural models	mNup107 complex dimer model, mNup50 structural model (Table S3)	Jumper et al., 2021, Mosalaganti et al., 2022, Sali et al., 1993, Varadi et al., 2022 and

			Eeswat et al., 2007[4,5,11–13]
	Bioinformatics information	Sequence information, coiled-coil propensities of the mTprs, orthologs alignments. Connectivity between residues/beads. Structural equivalent distances between ortholog Nups.	McDonnell et al., 2006[6]
	Immuno-EM	Upper and lower bound on the N-/C- terminus of mTprs	Frosst et al.,2002[14]
	Biochemical experiments including proximity labeling	Nuclear envelope binding domains of mNup153	Vollmer et al., 2015[15]

Table S2. Summary of the Integrative modeling of the yBasket. Related to Figures 3-5 and Figures S4-S6.

**1) Gathering data**	
Prior models	yNup84 complex dimer (PDB id: 7n84)[3]<br>Position of yNup84 complex derived by fitting on cryo-ET map using the chimera *fit map* feature[16,17].<br>yNup2 alphaFold model (AF-P32499-F1-model_v4.pdb) [4,5,18]<br>Generic Mlps coiled-coil segments models[19]
Physical principles and statistical preferences	Excluded volume<br>Sequence connectivity
Experimental information	**See Table S1**
**2) Representing the system**	
Sequences (Uniprot Ids)	yMlp1:Q02455, yMlp2:P40457, yMlp:Poly-ala of length yMlp1, yNup1:P20676, yNup2:P32499, yNup60:P39705, yNup120:P35729, yNup85:P46673, yNup145c:P49687, ySec13:Q04491, ySeh1:P53011, yNup84:P52891, yNup133:P36161
Composition (number of copies)	yMlp:2, yNup1:1, yNup2:2, yNup60:2, yNup120:2, yNup85:3, yNup145c:2, ySec13:2, ySeh1:2, yNup84:2, yNup133:2
Atomic (structured) components	yMlp.0: 71-183, 197-221, 239-281, 285-324, 340-371, 435-463, 535-570, 575-617, 622-668, 689-717, 745-773, 791-840, 844-893, 930-986, 990-1088, 1092-1127, 1143-1199, 1212-1251, 1254-1286, 1290-1339, 1343-1385, 1408-1457, yMlp.1: 71-183, 197-221, 239-281, 285-324, 340-371, 435-463, 535-570, 575-617, 622-668, 689-717, 745-773, 791-840, 844-893, 930-986, 990-1088, 1092-1127, 1143-1199, 1212-1251, 1254-1286, 1290-1339, 1343-1385, 1408-1457, yNup1.0: 1-32, 85-104, 106-123, yNup2.0: 83-136, 602-720, yNup2.1: 83-136, 602-720, yNup60.0: 27-47, 91-104, 106-119, 121-140, 142-162, yNup60.1: 27-47, 91-104, 106-119, 121-140, 142-162, yNup120.0: 1-29, 53-305, 311-711, 714-1036, yNup120.1: 1-29, 53-305, 311-711, 714-1036, yNup133.0: 63-183, 198-480, 490-763, 772-1155, yNup133.1: 56-77, 86-125, 133-144, 162-184, 193-200, 206-249, 258-480, 490-763, 772-1155, yNup145c.0: 119-712, yNup145c.1: 119-712, yNup84.0: 7-20, 27-80, 96-126, 136-364, 372-483, 506-562, 575-726, yNup84.1: 7-20, 27-80, 96-126, 136-364, 372-483, 506-562, 575-726, yNup85.0: 47-126, 132-230, 235-436, 451-744, yNup85.1: 47-126, 132-230, 235-436, 451-739, yNup85.2: 47-126, 132-230, 235-436, 451-739, ySec13.0: 8-157, 170-293, ySec13.1: 8-157, 170-293, ySeh1.0: 1-248, 288-346, ySeh1.1: 1-248, 288-346

Unstructured components	yMlp.0:-NTD: 1-70, yMlp.1:-NTD 1-70, yMlp.0:-LOOP: 184-196, 222-238, 282-284, 325-339, 372-434, 464-534, 571-574, 618-621, 669-688, 718-744, 774-790, 841-843, 894-929, 987-989, 1089-1091, 1128-1142, 1200-1211, 1252-1253, 1287-1289, 1340-1342, 1386-1407, yMlp.1:-LOOP: 184-196, 222-238, 282-284, 325-339, 372-434, 464-534, 571-574, 618-621, 669-688, 718-744, 774-790, 841-843, 894-929, 987-989, 1089-1091, 1128-1142, 1200-1211, 1252-1253, 1287-1289, 1340-1342, 1386-1407, yMlp.0:-CTD: 1458-1875, yMlp.1:-CTD: 1458-1875, yNup1.0: 33-84, 105-105, 124-335, yNup2.0: 51-82, yNup2.1: 51-82, yNup60.0: 1-26, 48-90, 105-105, 120-120, 141-141, 163-398, 505-539, yNup60.1: 1-26, 48-90, 105-105, 120-120, 141-141, 163-398, 505-539, yNup120.0: 30-52, 306-310, 712-713, 1037-103, yNup120.1: 30-52, 306-310, 712-713, 1037-1037, yNup133.0: 1-62, 184-197, 481-489, 764-771, 1156-1157, yNup133.1: 1-55, 78-85, 126-132, 145-161, 185-192, 201-205, 250-257, 481-489, 764-771, 1156-115, yNup145c.0: 1-118, yNup145c.1: 1-118, yNup84.0: 1-6, 21-26, 81-95, 127-135, 365-371, 484-505, 563-574, yNup84.1: 1-6, 21-26, 81-95, 127-135, 365-371, 484-505, 563-574, yNup85.0: 1-46, 127-131, 231-234, 437-450, yNup85.1: 1-46, 127-131, 231-234, 437-450, 740-744, yNup85.2: 1-46, 127-131, 231-234, 437-450, 740-744, ySec13.0: 1-7, 158-169, 294-297, ySec13.1: 1-7, 158-169, 294-297, ySeh1.0: 249-287, 347-349, ySeh1.1: 249-287, 347-349
Resolutions of structured components	1 [R1] residue per bead and 30 [R30] residue per bea
Resolutions of unstructured components	yMlp.0-NTD: 50 [R50] residue per bead<br>yMlp.1-NTD: 50 [R50] residue per bead<br>yMlp.0-LOOP: 5 [R5] residue per bead<br>yMlp.1-LOOP: 5 [R5] residue per bead<br>yMlp.0-CTD: 50 [R50] residue per bead<br>yMlp.1-CTD: 50 [R50] residue per bead<br>Rest all unstructured components 30 [R30] residue per bead
Structural coverage	77.39%
Rigid body (RB) definitions	RB1: yMlp.0 71-183, yMlp.1 71-183, RB2: yMlp.0 197-221, yMlp.1 197-221, RB3: yMlp.0 239-281, yMlp.1 239-281, RB4: yMlp.0 285-324, yMlp.1 285-324, RB5: yMlp.0 340-371, yMlp.1 340-371, RB6: yMlp.0 435-463,yMlp.1 435-463, RB7: yMlp.0 535-570, yMlp.1 535-570, RB8: yMlp.0 575-617, yMlp.1 575-617, RB9: yMlp.0 622-668, yMlp.1 622-668, RB10: yMlp.0 689-717, yMlp.1 689-717, RB11: yMlp.0 745-773, yMlp.1 745-773, RB12: yMlp.0 791-840, yMlp.1 791-840, RB13: yMlp.0 844-893, yMlp.1 844-893, RB14: yMlp.0 930-986, yMlp.1 930-986, RB15: yMlp.0 990-1088, yMlp.1 990-1088, RB16: yMlp.0 1092-1127, yMlp.1 1092-1127, RB17: yMlp.0 1143-1199, yMlp.1 1143-1199, RB18: yMlp.0 1212-1251, yMlp.1 1212-1251, RB19: yMlp.0 1254-1286, yMlp.1 1254-1286, RB20: yMlp.0 1290-1339, yMlp.1 1290-1339, RB21: yMlp.0 1343-1385, yMlp.1 1343-1385, RB22: yMlp.0 1408-1457, yMlp.1 1408-1457, RB23: yNup1.0 1-32, RB24: yNup1.0 85-104, RB25: yNup1.0 106-123, RB26: yNup2.0 83-136, RB27: yNup2.0 602-720, RB28: yNup2.1 83-136, RB29: yNup2.1 602-720, RB30: yNup60.0 27-47, RB31: yNup60.0 91-104, RB32: yNup60.0 106-119, RB33: yNup60.0 121-140, RB34: yNup60.0 142-162, RB35: yNup60.1 27-47, RB36: yNup60.1 91-104, RB37: yNup60.1 106-119, RB38: yNup60.1 121-140, RB39: yNup60.1 142-162, RB40: yNup120.0 1-29, yNup120.0 53-305, yNup120.0 311-711, yNup120.0 714-1036, RB41: yNup120.1 1-29, yNup120.1 53-305, yNup120.1 311-711, yNup120.1 714-1036, RB42: yNup85.0 47-126, yNup85.0 132-230, yNup85.0 235-436, yNup85.0 451-744, RB43: yNup85.1 47-126, yNup85.1 132-230, yNup85.1 235-436, yNup85.1 451-739, RB44: yNup85.2 47-126, yNup85.2 132-230, yNup85.2 235-436, yNup85.2 451-739, RB45: yNup145c.0 119-712, RB46: yNup145c.1 119-712, RB47: ySec13.0 8-157, ySec13.0 170-293, RB48: ySec13.1 8-157, ySec13.1 170-293, RB49: ySeh1.0 1-248, ySeh1.0 288-346, RB50: ySeh1.1 1-248, ySeh1.1 288-346, RB51: yNup84.0 7-20, yNup84.0 27-80, yNup84.0

	96-126, yNup84.0 136-364, yNup84.0 372-483, yNup84.0 506-562, yNup84.0 575-726, RB52:yNup84.1 7-20, yNup84.1 27-80, yNup84.1 96-126, yNup84.1 136-364, yNup84.1 372-483, yNup84.1 506-562, yNup84.1 575-726, RB53: yNup133.0 63-183, yNup133.0 198-480, yNup133.0 490-763, yNup133.0 772-1155, RB54: yNup133.1 56-77, yNup133.1 86-125, yNup133.1 133-144, yNup133.1 162-184, yNup133.1 193-200, yNup133.1 206-249, yNup133.1 258-480, yNup133.1 490-763, yNup133.1 772-1155
Spatial restraints encoded into scoring functions	Excluded volume; applied to the R30 representation<br>Sequence connectivity; applied to the R1 representation<br>Cross-link restraints; applied to the R1 representation
**3) Structural sampling**	
Sampling method	Replica Exchange Gibbs sampling, based on Metropolis Monte Carlo[20,21]
Replica exchange temperature range	1.0 - 4.0
Number of replicas	4
Number of Independent runs	300
Number of structures generated	15,467,933
Movers for flexible string of beads	Random translation up to 10 Å
Total time for sampling	180 hours on 1200 processors
**4) Validating the yBasket models**	
Models selected for validation	
Equilibrated models	2,335,171
Number of structures in samples A/B	All: 1,210,882 / 1,072,745
Number of structures selected for RMSD clustering sample A/B	Random sub-sample: 7,857 / 7,987
p-value of non-parametric Kolmogorov-Smirnov two-sample test	0.0556 (threshold p-value > 0.05)
Kolmogorov-Smirnov two-sample test statistic, D	0.0
Throughness of the structural sampling	
Sampling precision	70.41 Å
Homogeneity of proportions χ^2-test (p-value)/Cramers V value	0.0/0.095 (thresholds: p-value<0.05 OR Cramer's V<0.1)
Number of clusters	5
Cluster populations	Cluster 1 : 93.5%, Cluster 2 : 4.0%, Cluster 3 : 1.2%, Cluster 4 : 1.0%, Cluster 5 : 0.3%
Cluster precisions	Cluster 1 : 57.2 Å, Cluster 2 : 58.6 Å, Cluster 3 : 56.5 Å, Cluster 4 : 53.3 Å, Cluster 5 : 59.8 Å

Average cross-correlation between localization probability densities of samples A and B	0.96
Validation by information used for modeling	
Percent of sequence connectivity restraints satisfied by ensemble	99%
Percent Excluded volume restraint satisfied by ensemble	Mean value 99.2% at R[30] resolution and mean value 99.9% at R[1] resolution
Cross-correlation between localization probability densities and cryo-ET map	0.82
Percent crosslink restraint satisfied by ensemble	94%
**5) Software and data availability**	
Software	
Modeling programs	IMP PMI module, version-a41075a<br>Integrative Modeling Platform (IMP), version 2.19 (https://integrativemodeling.org)[22]<br>gmconvert[23]
Modeling scripts	https://github.com/neeleshsoni21/Yeast_NPC_Basket
Structure prediction	AlphaFold2
Visualization and plotting	UCSF Chimerax
Data	
PDB-dev accession code	Collection of all models (PDBDEV: PDBDEV_G_1000004) and Yeast NPC (PDBDEV: PDBDEV_00000386, DBDEV: PDBDEV_00000387).

Table S3. Summary of the Integrative modeling of the mBasket. Related to Figures 3-5 and Figures S4-S6.

**1) Gathering data**	
Prior models	mNup107 complex dimer comparative model built on Human Nup107 complex (PDB id: 7r5j)[11–13]<br>Position of yNup107 complex derived by fitting on cryo-ET map using the chimera *fit map* feature[16,17].<br>yNup50 alphaFold model (AF-Q9JIH2-F1-model_v4.pdb) [4,5,18]<br>Tpr coiled-coil segments models[19]
Physical principles and statistical preferences	Excluded volume<br>Sequence connectivity
Experimental information	**See Table S1**
**2) Representing the system**	
Sequences (Uniprot Ids)	mTpr:F6ZDS4, mNup50:Q9JIH2, mNup153:E9Q3G8,

	mNup43:P59235, mSeh1:Q8R2U0, mNup98-mNup96:Q6PFD9, mNup85:Q8R480, mNup37:Q9CWU9, mNup107:Q8BH74, mSec13:Q9D1M0, mNup133:Q8R0G9, mNup160:Q9Z0W3
Composition (number of copies)	mTpr: 2, mNup50: 2, mNup153: 2, mNup107: 2, mNup133: 2, mNup160: 2, mNup37: 2, mNup43: 2, mNup85: 2, mNup96: 2, mSec13: 2, mSeh1: 2
Atomic (structured) components	mTpr.0: 96-120, 124-180, 187-247, 254-285, 289-356, 360-405, 413-451, 511-595, 639-681, 757-883, 901-946, 947-999, 1004-1060, 1064-1106, 1107-1131, 1135-1163 1170-1201, 1205-1254, 1281-1337, 1343-1420, 1424-1491, 1543-1616, 1627-1690, mTpr.1: 96-120, 124-180, 187-247, 254-285, 289-356, 360-405, 413-451, 511-595, 639-681, 757-883, 901-946, 947-999, 1004-1060, 1064-1106, 1107-1131, 1135-1163, 1170-1201, 1205-1254, 1281-1337, 1343-1420, 1424-1491, 1543-1616, 1627-1690, mNup50.0: 151-204, 355-466, mNup50.1: 151-204, 355-466, mNup153.0: 36-57, mNup153.1: 36-57, mNup107.0: 145-926, mNup107.1: 145-926, mNup133.0: 70-1155, mNup133.1: 70-1155, mNup160.0: 1-1402, mNup160.1: 1-1402, mNup37.0: 1-326, mNup37.1: 1-326, mNup43.0: 1-292, 327-380, mNup43.1: 1-292, 327-380, mNup85.0: 1-656, mNup85.1: 1-656, mNup96.0: 1111-1159, 1194-1816, mNup96.1: 1111-1159, 1194-1816, mSec13.0: 1-302, mSec13.1: 1-302, mSeh1.0: 1-324, mSeh1.1: 1-324
Unstructured components	mTpr.0:-NTD: 1-95, mTpr.1:-NTD: 1-95, mTpr.0:-LOOP: 121-123, 181-186, 248-253, 286-288, 357-359, 406-412, 452-510, 596-638, 682-756, 884-900, 1000-1003, 1061-1063, 1132-1134, 1164-1169, 1202-1204, 1255-1280, 1338-1342, 1421-1423, 1492-1542, 1617-1626, mTpr.1:-LOOP: 1-95, 121-123, 181-186, 248-253, 286-288, 357-359, 406-412, 452-510, 596-638, 682-756, 884-900, 1000-1003, 1061-1063, 1132-1134, 1164-1169, 1202-1204, 1255-1280, 1338-1342, 1421-1423, 1492-1542, 1617-1626, mTpr.0:-CTD: 1691-2431, mTpr.1:-CTD: 1691-2431, mNup50.0: 1-150, 205-354, mNup50.1: 1-150, 205-354, mNup153.0: 1-35, 58-428, 540-574, mNup153.1: 1-35, 58-428, 540-574
Resolutions of structured components	1 [R1] residue per bead and 30 [R30] residue per bead
Resolutions of unstructured components	mTpr.0-NTD: 50 [R50] residue per bead<br>mTpr.1-NTD: 50 [R50] residue per bead<br>mTpr.0-LOOP: 30 [R5] residue per bead<br>mTpr.1-LOOP: 30 [R5] residue per bead<br>mTpr.0-CTD: 50 [R50] residue per bead<br>mTpr.1-CTD: 50 [R50] residue per bead<br>Rest all unstructured components 30 [R30] residue per bead
Structural coverage	79.48%
Rigid body (RB) definitions	RB1: mTpr.0 96-120, mTpr.1 96-120, RB2: mTpr.0 124-180, mTpr.1 124-180, RB3: mTpr.0 187-247, mTpr.1 187-247, RB4: mTpr.0 254-285, mTpr.1 254-285, RB5: mTpr.0 289-356, mTpr.1 289-356, RB6: mTpr.0 360-405, mTpr.1 360-405, RB7: mTpr.0 413-451, mTpr.1 413-451, RB8: mTpr.0 511-595, mTpr.1 511-595, RB9: mTpr.0 639-681, mTpr.1 639-681, RB10: mTpr.0 757-883, mTpr.1 757-883, RB11: mTpr.0 901-946, mTpr.1 901-946, RB12: mTpr.0 947-999, mTpr.1 947-999, RB13: mTpr.0 1004-1060, mTpr.1 1004-1060, RB14: mTpr.0 1064-1106, mTpr.1 1064-1106, RB15: mTpr.0 1107-1131, mTpr.1 1107-1131, RB16: mTpr.0 1135-1163, mTpr.1 1135-1163, RB17: mTpr.0 1170-1201, mTpr.1 1170-1201, RB18: mTpr.0 1205-1254, mTpr.1 1205-1254, RB19: mTpr.0 1281-1337, mTpr.1 1281-1337, RB20: mTpr.0 1343-1420, mTpr.1 1343-1420, RB21: mTpr.0 1424-1491, mTpr.1 1424-1491, RB22: mTpr.0 1543-1616, mTpr.1 1543-1616, RB23: mTpr.0 1627-1690, mTpr.1 1627-1690, RB24: mNup50.0 151-204, RB25: mNup50.0 355-466, RB26: mNup50.1 151-204, RB27: mNup50.1 355-466, RB28: mNup153.0

	36-57, RB29: mNup153.1 36-57<br><br>Fixed RB components:<br>RB30: mNup160.0 1-1402, RB31: mNup160.1 1-1402, RB32: mNup85.0 1-656, RB33: mNup85.1 1-656, RB34: mNup96.0 1111-1159, mNup96.0 1194-1816, RB35: mNup96.1 1111-1159, mNup96.1 1194-1816, RB36: mSec13.0 1-302, RB37: mSec13.1 1-302, RB38: mSeh1.0 1-324, RB39: mSeh1.1 1-324, RB40: mNup107.0 145-926, RB41: mNup107.1 145-926, RB42: mNup133.0 70-1155, RB43: mNup133.1 70-1155, RB44: mNup37.0 1-326, RB45: mNup37.1 1-326, RB46: mNup43.0 1-292, mNup43.0 327-380, RB47: mNup43.1 1-292, mNup43.1 327-380
Spatial restraints encoded into scoring functions	Excluded volume; applied to the R30 representation<br>Sequence connectivity; applied to the R1 representation<br>Cross-link restraints; applied to the R1 representation
**3) Structural sampling**	
Sampling method	Replica Exchange Gibbs sampling, based on Metropolis Monte Carlo [20,21]
Replica exchange temperature range	1.0 - 4.0
Number of replicas	2
Number of Independent runs	200
Number of structures generated	9,569,309
Movers for flexible string of beads	Random translation up to 10 Å
Total time for sampling	168 hours on 400 processors
**4) Validating the mBasket models**	
Models selected for validation	
Equilibrated models	1,216,228
Number of structures in samples A/B	All: 520,767 / 474,386
Number of structures selected for RMSD clustering sample A/B	Random sub-sample: 6,886 / 7,783
p-value of non-parametric Kolmogorov-Smirnov two-sample test	0.056 (threshold p-value > 0.05)
Kolmogorov-Smirnov two-sample test statistic, D	0.0
Throughness of the structural sampling	
Sampling precision	52.9 Å
Homogeneity of proportions χ^2-test (p-value)/Cramers V value	0.0/0.097 (thresholds: p-value<0.05 OR Cramer's V<0.1)
Number of clusters	4

Cluster populations	Cluster 1 : 75.6%, Cluster 2 : 15.6%, Cluster 3 : 8.1%, Cluster 4 : 0.7%
Cluster precisions	Cluster 1 : 41.9 Å, Cluster 2 : 34.9 Å, Cluster 3 : 41.2 Å, Cluster 4 : 31.4 Å
Average cross-correlation between localization probability densities of samples A and B	0.95
Validation by information used for modeling	
Percent of sequence connectivity restraints satisfied by ensemble	99%
Percent Excluded volume restraint satisfied by ensemble	Mean value 94.55% at R[30] resolution and mean value 99.63% at R[1] resolution
Cross-correlation between localization probability density and cryo-ET map	0.87
**5) Software and data availability**	
Software	
Modeling programs	IMP PMI module, version-a41075a<br>Integrative Modeling Platform (IMP), version 2.19 (https://integrativemodeling.org) [22]<br>Gmconvert [23]
Modeling scripts	https://github.com/neeleshsoni21/Mouse_NPC_Basket
Structure prediction	AlphaFold2
Visualization and plotting	UCSF Chimerax
Data	
PDB-dev accession code	Collection of all models (PDBDEV: PDBDEV_G_1000004) and Mammalian NPC (PDBDEV: PDBDEV_00000384, PDBDEV: PDBDEV_00000385)

References

1. Kim, S.J., Fernandez-Martinez, J., Nudelman, I., Shi, Y., Zhang, W., Raveh, B., Herricks, T., Slaughter, B.D., Hogan, J.A., Upla, P., et al. (2018). Integrative structure and functional anatomy of a nuclear pore complex. Nature *555*, 475–482.

2. Niepel, M., Molloy, K.R., Williams, R., Farr, J.C., Meinema, A.C., Vecchietti, N., Cristea, I.M., Chait, B.T., Rout, M.P., and Strambio-De-Castillia, C. (2013). The nuclear basket proteins Mlp1p and Mlp2p are part of a dynamic interactome including Esc1p and the proteasome. Mol. Biol. Cell *24*, 3920–3938.

3. Akey, C.W., Singh, D., Ouch, C., Echeverria, I., Nudelman, I., Varberg, J.M., Yu, Z., Fang, F., Shi, Y., Wang, J., et al. (2022). Comprehensive structure and functional adaptations of the yeast nuclear pore complex. Cell *185*, 361–378.e25.

4. Jumper, J., Evans, R., Pritzel, A., Green, T., Figurnov, M., Ronneberger, O., Tunyasuvunakool, K., Bates, R., Žídek, A., Potapenko, A., et al. (2021). Highly accurate protein structure prediction with AlphaFold. Nature *596*, 583–589.

5. Varadi, M., Anyango, S., Deshpande, M., Nair, S., Natassia, C., Yordanova, G., Yuan, D., Stroe, O., Wood, G., Laydon, A., et al. (2022). AlphaFold Protein Structure Database: massively expanding the structural coverage of protein-sequence space with high-accuracy models. Nucleic Acids Res. *50*, D439–D444.

6. McDonnell, A.V., Jiang, T., Keating, A.E., and Berger, B. (2006). Paircoil2: improved prediction of coiled coils from sequence.

Bioinformatics *22*, 356–358.

7. Cibulka, J., Bisaccia, F., Radisavljević, K., Gudino Carrillo, R.M., and Köhler, A. (2022). Assembly principle of a membrane-anchored nuclear pore basket scaffold. Sci Adv *8*, eabl6863.

8. Mészáros, N., Cibulka, J., Mendiburo, M.J., Romanauska, A., Schneider, M., and Köhler, A. (2015). Nuclear pore basket proteins are tethered to the nuclear envelope and can regulate membrane curvature. Dev. Cell *33*, 285–298.

9. Li, Y., Aksenova, V., Tingey, M., Yu, J., Ma, P., Arnaoutov, A., Chen, S., Dasso, M., and Yang, W. (2021). Distinct roles of nuclear basket proteins in directing the passage of mRNA through the nuclear pore. Proc. Natl. Acad. Sci. U. S. A. *118*. https://doi.org/10.1073/pnas.2015621118.

10. Ori, A., Banterle, N., Iskar, M., Andrés-Pons, A., Escher, C., Khanh Bui, H., Sparks, L., Solis-Mezarino, V., Rinner, O., Bork, P., et al. (2013). Cell type-specific nuclear pores: a case in point for context-dependent stoichiometry of molecular machines. Mol. Syst. Biol. *9*, 648.

11. Mosalaganti, S., Obarska-Kosinska, A., Siggel, M., Taniguchi, R., Turoňová, B., Zimmerli, C.E., Buczak, K., Schmidt, F.H., Margiotta, E., Mackmull, M.-T., et al. (2022). AI-based structure prediction empowers integrative structural analysis of human nuclear pores. Science *376*, eabm9506.

12. Sali, A., and Blundell, T.L. (1993). Comparative protein modelling by satisfaction of spatial restraints. J. Mol. Biol. *234*, 779–815.

13. Eswar, N., Webb, B., Marti-Renom, M.A., Madhusudhan, M.S., Eramian, D., Shen, M.-Y., Pieper, U., and Sali, A. (2007). Comparative protein structure modeling using MODELLER. Curr. Protoc. Protein Sci. *Chapter 2*, Unit 2.9.

14. Frosst, P., Guan, T., Subauste, C., Hahn, K., and Gerace, L. (2002). Tpr is localized within the nuclear basket of the pore complex and has a role in nuclear protein export. J. Cell Biol. *156*, 617–630.

15. Vollmer, B., Lorenz, M., Moreno-Andrés, D., Bodenhöfer, M., De Magistris, P., Astrinidis, S.A., Schooley, A., Flötenmeyer, M., Leptihn, S., and Antonin, W. (2015). Nup153 Recruits the Nup107-160 Complex to the Inner Nuclear Membrane for Interphasic Nuclear Pore Complex Assembly. Dev. Cell *33*, 717–728.

16. Goddard, T.D., Huang, C.C., Meng, E.C., Pettersen, E.F., Couch, G.S., Morris, J.H., and Ferrin, T.E. (2018). UCSF ChimeraX: Meeting modern challenges in visualization and analysis. Protein Sci. *27*, 14–25.

17. Pettersen, E.F., Goddard, T.D., Huang, C.C., Couch, G.S., Greenblatt, D.M., Meng, E.C., and Ferrin, T.E. (2004). UCSF Chimera--a visualization system for exploratory research and analysis. J. Comput. Chem. *25*, 1605–1612.

18. Kohn, J.E., Millett, I.S., Jacob, J., Zagrovic, B., Dillon, T.M., Cingel, N., Dothager, R.S., Seifert, S., Thiyagarajan, P., Sosnick, T.R., et al. (2004). Random-coil behavior and the dimensions of chemically unfolded proteins. Proc. Natl. Acad. Sci. U. S. A. *101*, 12491–12496.

19. Soni, N., and Madhusudhan, M.S. (2024). COCONUT: An analysis of coiled-coil regions in proteins. bioRxiv. https://doi.org/10.1101/2024.03.25.586698.

20. Shi, Y., Pellarin, R., Fridy, P.C., Fernandez-Martinez, J., Thompson, M.K., Li, Y., Wang, Q.J., Sali, A., Rout, M.P., and Chait, B.T. (2015). A strategy for dissecting the architectures of native macromolecular assemblies. Nat. Methods *12*, 1135–1138.

21. Swendsen, R.H., and Wang, J.S. (1986). Replica Monte Carlo simulation of spin glasses. Phys. Rev. Lett. *57*, 2607–2609.

22. Russel, D., Lasker, K., Webb, B., Velázquez-Muriel, J., Tjioe, E., Schneidman-Duhovny, D., Peterson, B., and Sali, A. (2012). Putting the pieces together: integrative modeling platform software for structure determination of macromolecular assemblies. PLoS Biol. *10*, e1001244.

23. Kawabata, T. (2008). Multiple subunit fitting into a low-resolution density map of a macromolecular complex using a gaussian mixture model. Biophys. J. *95*, 4643–4658.
