# Supplementary material for: The molecular architecture of the nuclear basket
Source: Cell. Author manuscript; Available in PMC 2024 Sep 22. (PMC11416316; doi:10.1016/j.cell.2024.07.020)
Supplement: 3 [file NIHMS2016536-supplement-3.pdf]

Supplemental figures

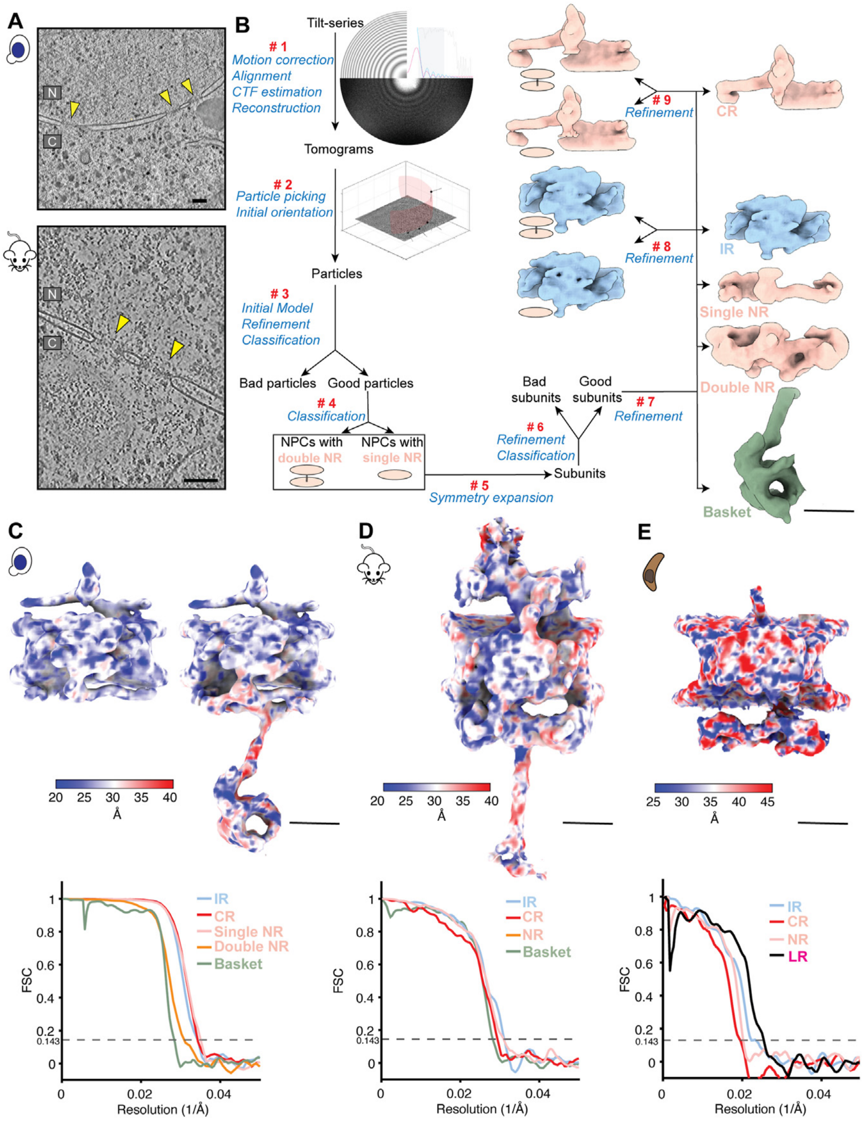

Figure S1. Workflow of the subtomogram analysis of the NPC, related to Figure 1

(A) Slices of representative tomograms from yeast, mammalian cells, and *T. gondii*, where nuclear pores are indicated by yellow arrows in the nucleus. Scale bars, 100 nm.

(B) Processing workflow of the subtomogram analysis of the NPC, where steps are indicated by #. The workflow is similar for data from all organisms. Some steps, like #4 for classifying out yNPCs with single and double NR, are more specific for yeast. For yeast, maps of the subunit of IR and CR from NPCs with single and double NR, as shown, resulting from steps #8 and #9 are similar. The same workflow was used for mNPC and pNPC. Scale bars, 200 Å. All the maps and subsequent models shown in the manuscript, except Figures 2A and S3C, were ultimately determined at the level of a single subunit, without using any symmetrization. Focused refinements of the different rings within a subunit were combined into a composite map representing an entire single subunit of the NPC and its basket, which were then C8-symmetrized to depict the whole NPC (see STAR Methods).

(C–E) Local resolution maps of the subunit of the NPC (top) along with Fourier shell correlation (FSC) curves of subunits of different rings (bottom) for yeast (C), mammalian (D), and *T. gondii* (E). Scale bars, 200 Å. CR, cytoplasmic ring; IR, inner ring; NR, nuclear ring; NE, nuclear envelope; LR, lumenal ring.

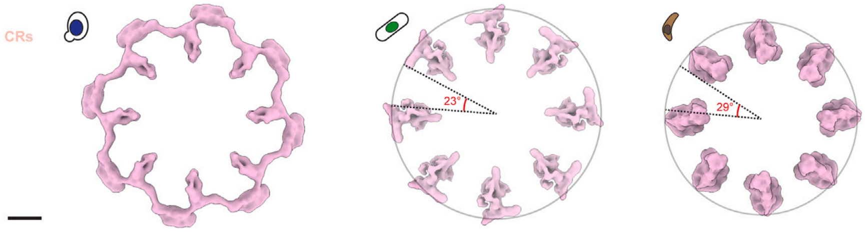

Figure S2. CRs of NPCs from different organisms can have differing extents of completeness, related to Figure 1
The circumference not covered (in central angles) by CRs of *S. cerevisiae* (left), *S. pombe* (middle; EMDB: 11373[44]), and *T. gondii* (right) is 0° (100% – 0% = 100% covered), 23° × 8 (100% – 51% = 49% covered), and 29° × 8 (100% – 64% = 36% covered), respectively. Scale bars, 200 Å.

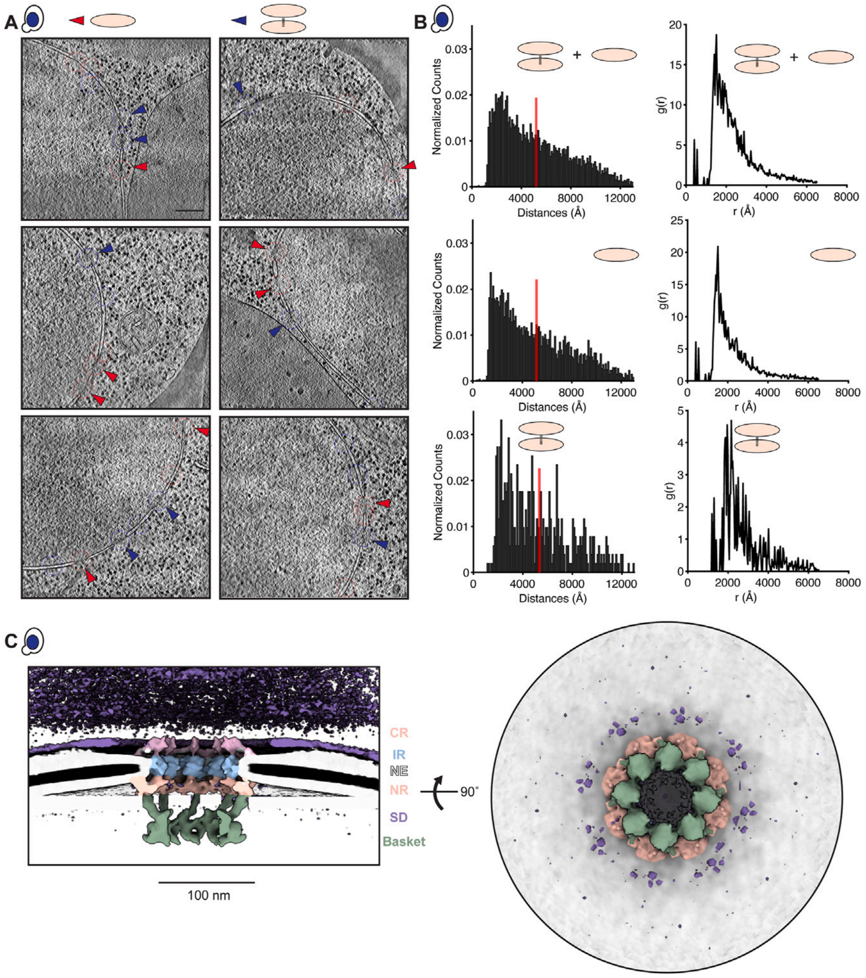

Figure S3. yNPCs have less pronounced nuclear surrounding densities, and their single and double NR variants are similarly distributed across the nucleus, related to Figure 2

(A) Slices of representative tomograms from yeast cells highlight yNPCs with single (indicated by red arrows) and double NRs (blue arrows). These images demonstrate that yNPCs with single and double NRs with a stable basket can be adjacent rather than in distinctly separate areas. Scale bars, 100 nm.

(B) Analysis of the yNPC's pairwise distance distribution (left) and the radial distribution function [g(r)] (right), encompassing all NPCs, as well as subsets with either single or double NRs, reveals a similar spatial distribution across the nucleus for all yNPCs. This is evidenced by the qualitative similarity in these distributions.

(C) The cross-sectional (left) and nucleoplasmic view (right) of the map of yNPC shows the extent of crowding around the yNPC, with the surrounding densities (SDs) shown at a very low isosurface threshold. These densities, compared with their counterpart in mNPCs, are much weaker.

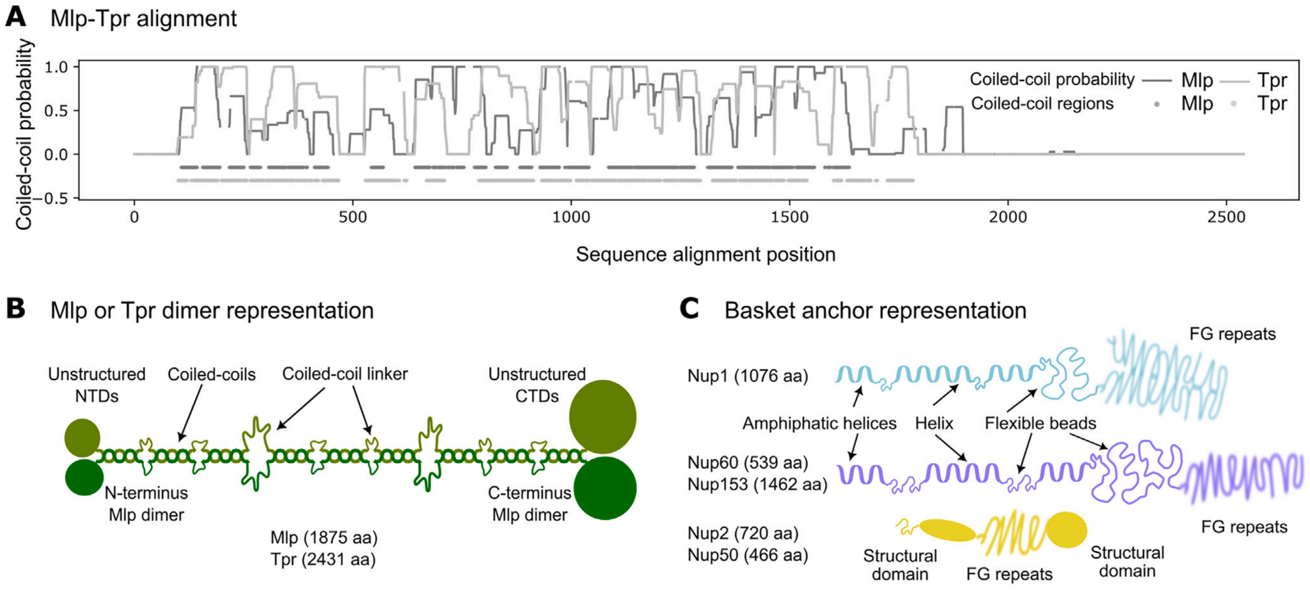

Figure S4. Nuclear basket model representation, related to Figure 3

(A) Coiled-coil probabilities of yMlp (dark gray) and mTpr (light gray) at different positions in their sequence. The region represented as coiled-coil segments is shown as horizontal dots in a straight line below 0.0.

(B) Schematic representation of a yMlp or mTpr dimer. Coiled-coil regions were represented as rigid bodies and linked through flexible strings of beads. The unstructured N- and C-terminal domains (NTDs and CTDs, respectively) were represented as flexible strings of beads.

(C) Schematic representation of the FG NR anchor Nups: yNup1, yNup2/mNup50, yNup60/mNup153. All structural regions, including amphipathic helices (AHs) at the N terminus, were represented as rigid helices; all non-structural regions, excluding FG repeats, were represented as flexible strings of beads. FG repeats were not included in the model. The number of amino acid (aa) residues for different Nups are indicated in parenthesis.

CellPress
OPEN ACCESS

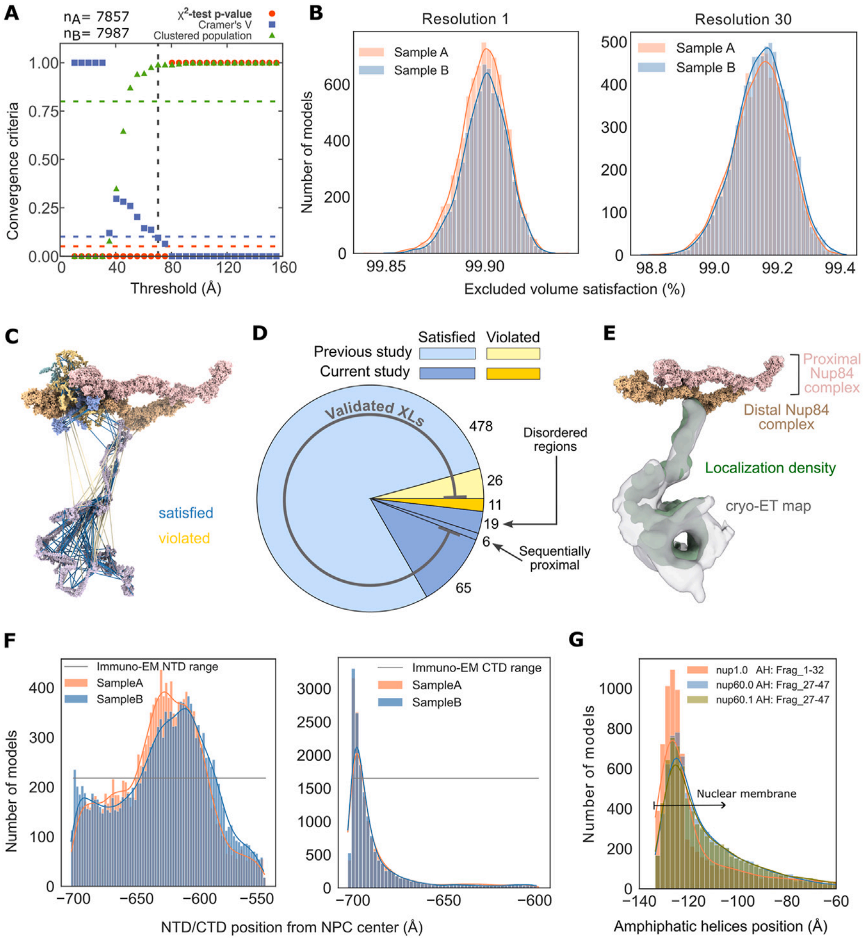

Figure S5. Validation of integrative models of the yBasket, related to Figures 4 and 5

(A) Criteria for determining the sampling precision (y axis) were evaluated as a function of the RMSD clustering threshold (x axis) (n = 15,844 models). First, the p value is computed using the χ^2 test (one-sided) for homogeneity of proportions (red dots). Second, an effect size for the χ^2 test is quantified by the Cramers's V value (blue squares). Third, the population of the structures in sufficiently large clusters is shown as green triangles. The vertical dotted gray line indicates the RMSD clustering threshold at which three conditions are satisfied (Cramer's V (0.095) < 0.1 (blue horizontal dotted lines), p value (0.0) < 0.05 and the population of clustered structures (0.99) > 0.8 (green horizontal dotted lines, thus defining the sampling precision of 70.4 Å. The three solid curves (red, blue, and green) were drawn through the points to help visualize the results. The number of models in two randomly drawn samples, sample A and sample B, from a pool of filtered models (n = 2,283,627 models) is denoted by nA (7,857 models) and nB (7,987 models).

(B) Histogram of excluded volume restraint satisfaction of samples nA and nB at two different resolutions of 1 and 30 residues per bead.

(C) Chemical crosslinks were mapped onto the structure of the yBasket subunit with lines. Satisfied crosslinks where Euclidian Cα–Cα distances below 35 Å in the model ensemble are represented with blue lines, whereas violated crosslinks are with yellow lines.

(D) A pie chart of satisfied (shades of blue) and violated (shades of yellow) crosslinks grouped into previously published[6] (light blue and light yellow) and this study (dark blue and dark yellow) crosslinks. The validated crosslinks (gray arc) account for 94% of the crosslinks; validated crosslinks have MS2 spectra with multiple backbone fragmentations of both peptides and peptides of at least 6 residues. Of the non-validated crosslinks, 11 were violated, 6 were trivially satisfied due to sequence proximity, and 19 were in the disordered/N terminus/C terminus regions of the basket distal density of our model.

(E) A side view of the yBasket models localization density attached to the distal yNup84 complex spans most of the cryo-ET density map.

(F) Satisfaction of experimentally derived range (gray line) of the position of yMlps N and C termini by the model ensemble derived values (coral and light blue).

(G) Satisfaction of experimentally derived range (gray line) of the position of amphipathic transmembrane helices of yNup1 and yNup60 by the model ensemble derived values (coral and light blue).

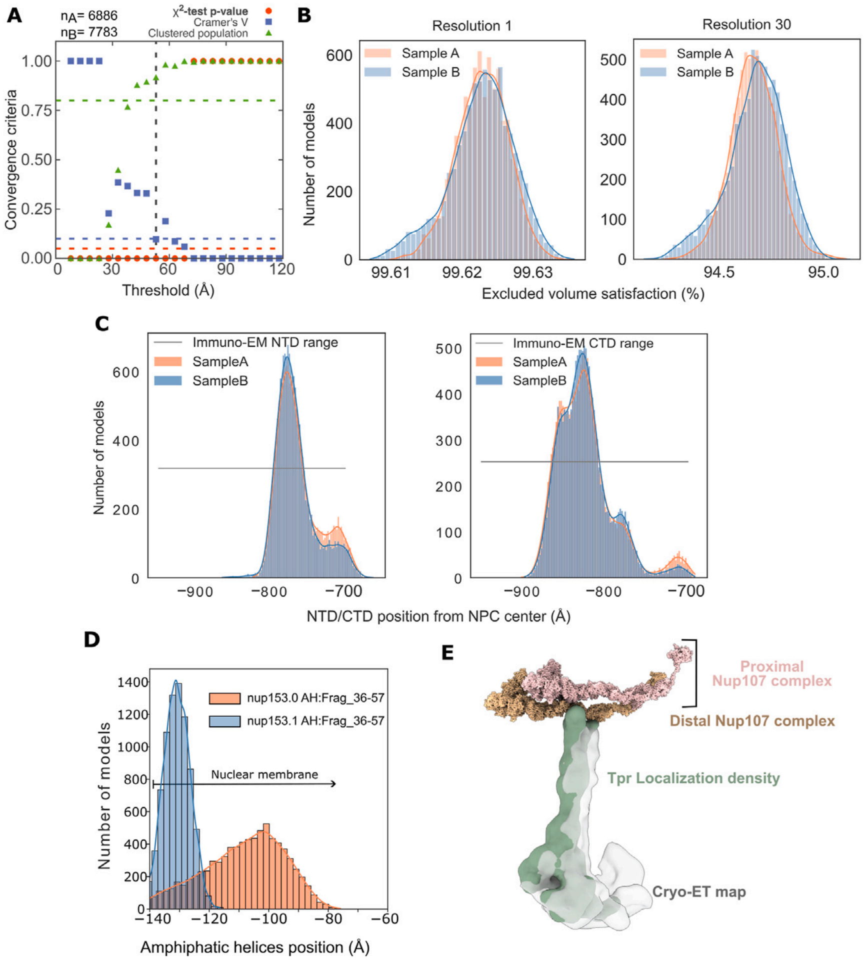

Figure S6. Validation of integrative models of the mBasket, related to Figures 4 and 5

(A) Criteria for determining the sampling precision (y axis) were evaluated as a function of the RMSD clustering threshold (x axis) (*n* = 14,669 models). First, the *p* value is computed using the χ^2 test (one-sided) for homogeneity of proportions (red dots). Second, an effect size for the χ^2 test is quantified by the Cramers's V value (blue squares). Third, the population of the structures in sufficiently large clusters is shown as green triangles. The vertical dotted gray line indicates the RMSD clustering threshold at which three conditions are satisfied: Cramer's V (0.097) < 0.1 (blue horizontal dotted lines), *p* value (0.0) < 0.05, and the population of clustered structures (0.92) > 0.8 (green horizontal dotted lines), thus defining the sampling precision of 52.9 Å. The three solid curves (red, blue, and green) were drawn through the points to help visualize the results. The number of models in two randomly drawn samples from a pool of filtered models (*n* = 995,151 models) is denoted by nA (6,886 models) and nB (7,783 models).

(B) Histogram of excluded volume restraint satisfaction of samples nA and nB at two different resolutions of 1 and 30 residues per bead.

(C) Satisfaction of experimentally derived range (gray line) of the position of mTpr's N and C termini by the model ensemble derived values (coral and light blue).

(D) Satisfaction of the experimentally derived range (gray line) of the position of amphipathic transmembrane helices of mNup153 by the model ensemble derived values (coral and light blue).

(E) A side view of the mBasket models localization density attached to the distal mNup107 complex spans most of the cryo-ET density map.
